# On chip control and detection of complex SPP and waveguide modes based on plasmonic interconnect circuits

**DOI:** 10.1515/nanoph-2024-0298

**Published:** 2024-09-09

**Authors:** Canran Zhang, Yijing Xu, Hui Tao, Pan Wang, Yunkang Cui, Qilong Wang

**Affiliations:** Joint International Research Laboratory of Information Display and Visualization, School of Electronic Science and Engineering, Southeast University, Nanjing 210096, China; Department of Mathematics and Physics, Nanjing Institute of Technology, Nanjing 211167, China

**Keywords:** surface plasmon polaritons, optical near fields, propagating light fields, plasmonic interconnect circuits, mode detection

## Abstract

Optical interconnects, leveraging surface plasmon modes, are revolutionizing high-performance computing and AI, overcoming the limitations of electrical interconnects in speed, energy efficiency, and miniaturization. These nanoscale photonic circuits integrate on-chip light manipulation and signal conversion, marking significant advancements in optoelectronics and data processing efficiency. Here, we present a novel plasmonic interconnect circuit, by introducing refractive index matching layer, the device supports both pure SPP and different hybrid modes, allowing selective excitation and transmission based on light wavelength and polarization, followed by photocurrent conversion. We optimized the coupling gratings to fine-tune transmission modes around specific near-infrared wavelengths for effective electrical detection. Simulation results align with experimental data, confirming the device’s ability to detect complex optical modes. This advancement broadens the applications of plasmonic interconnects in high-speed, compact optoelectronic and sensor technologies, enabling more versatile nanoscale optical signal processing and transmission.

## 1 Introduction

Against the backdrop of the increasing demand for computing power in Artificial Intelligence (AI) and Big Data, optical interconnects for future data centers and high-performance computing platforms have become a research focus to overcome the speed, energy efficiency, manufacturing cost and other issues faced by traditional electrical interconnects [1]–[7]. However, miniaturization of functional photonic interconnect circuits (PICs) is hindered by diffraction limit. Fortunately, surface plasmon modes supported by noble-meta structures can constrain light at subwavelength scale, providing the realization of nanoscale PICs [8], [9], [10], [11]. Numerous plasmonic structures and devices, including on-chip light sources [12]–[19], passive waveguides [20]–[24], modulators [25]–[30], and detectors [31]–[37], have undergone investigation. These advancements align with and enhance the progression of silicon-based optoelectronics and optical interconnect technology, significantly boosting the efficiency and capabilities of integrated optoelectronic devices [38]–[42]. The proposed plasmonic interconnect circuits, built on the aforementioned foundation, integrate on-chip optical field excitation, transmission/modulation, and detection. They showcase formidable capabilities in on-chip optical field manipulation, bandwidth expansion, footprint minimization, and power consumption [24], [43], [44], [45], [46].

For a complete plasmonic interconnect circuit, surface plasmon couplers are crucial components that not only enable efficient excitation of surface plasmon polariton (SPP) but also facilitate precise multi-dimensional control of optical parameters [47], [48]. The surface plasmon waveguides connect to the couplers, ensuring that the optical field carrying information is transmitted faithfully and efficiently [49], [50], [51], and provide a platform for indirect modulation methods such as electro-optic [25], [27], thermo-optic [52], and magneto-optic [53]. The surface plasmon detectors are responsible for efficiently and rapidly converting plasmon signals into electrical signals for interfacing with external circuits [32], [33], [34], [36], [44], [45]. The above components demonstrate the continuous transformation process from the optical near-field to the propagating field and then to the electrical signal.

On the one hand, the optical near-field, sensitive to the properties of excitation light and brought about by plasmonic structures, provides rich available dimensions for bandwidth enhancement [47], [49], [54], [55]. On the other hand, plasmonic interconnect circuits may also provide a possible technical approach for on-chip detection of optical near-field, as generally observing optical near-field or mode often requires bulky external instruments, such as near-field scanning optical microscopy (NSOM) and angle-resolved spectroscopy (ARS) [50], [56], [57]. The transition from optical near-field interactions to propagation field can significantly broaden the application scope of integrated photonics [47], [55]. Then, combining the optical information generated in the above process with SPP detectors for electrical readout truly establishes a bridge between integrated photonics and microelectronics, which aligns with the long-standing expectations people have harbored for plasmonic devices [1], [11], [40].

In this paper, we constructed a classic plasmonic interconnect circuit based on the subwavelength metal grating surface plasmon coupler and metal–semiconductor–metal (MSM) photodetector coupled to a metal waveguide. It must be emphasized that our previous work utilized a basic asymmetric grating coupler design to achieve unidirectional transmission of SPP, this is an improvement in device performance [58]. This paper extends the functional applications of this plasmonic optoelectronic interconnect platform.

Due to the SiO_2_ refractive index matching layer covering the metal plasmonic structure, the plasmonic interconnect circuit that we constructed supports not only pure SPP transmission mode but also multiple hybrid modes. These modes greatly expand the applicable wavebands of the devices, among which the hybrid plasmonic mode can combine the high localization of traditional plasmonic modes and the low loss characteristic of dielectric modes, representing the future direction of development for plasmonic interconnect circuits [40]. These modes can be selectively excited and transmitted by incident light of different wavelengths and polarization states and then converted into photocurrent using MSM photodetector, enabling in-plane control and electrical detection of the optical modes. This functionality was unattainable with previous plasmonic devices and represents a novel operating mode. In order to improve the signal transmission efficiency and expand the differences between different modes, we used particle swarm optimization (PSO) algorithm to optimize the structural parameters of the coupling gratings, and ultimately locked the different optical transmission modes in the vicinity of three commonly used near-infrared bands (980 nm, 1,064 nm, 1,310 nm) for practical electrical detection. The simulated band structure of the coupling gratings and the electric field distribution at the coupling gratings and waveguide clearly reflect the source of different optical transmission modes and the evolution process from the optical near field to the propagation field. The actual device’s photocurrent test results at different wavelengths and polarization states are basically consistent with the simulation prediction, verifying the electrical detection of multiple optical transmission modes. Our work enhances the potential applications of plasmonic interconnect circuits in high-speed, miniaturized optoelectronic or sensor devices, offering a more flexible and efficient platform for optical signal transmission and processing at the nanoscale.

## 2 Methods

### 2.1 Numerical simulations

The numerical simulation of the coupling/decoupling efficiency and electric field distribution in this work were carried out via finite-difference time-domain (FDTD) method (FDTD solution, Lumerical Inc., Vancouver, Canada). The simulation is two-dimensional (2D) because the model is symmetric in the *y* direction. The structure is surrounded by air (*n*_air_ = 1), and the temperature is 300 K. The simulation time is set to 1,000 fs and the overall mesh size is 5 nm × 5 nm. In the simulation model of coupling efficiency and electric field distribution, the perfectly matched layer (PML) boundary condition is used in both the *x* direction and the *z* direction. The incident total-field-scattered-field (TFSF) source illuminates the coupling gratings vertically from the top side of the structure (The red dashed box in Figures 2a and S3a). A power monitor placed above the waveguide at a distance of 1 μm from the end of the gratings is used to calculate coupling efficiency and eliminate the influence of the incident light [59], [60] and a power monitor at the interface between the decoupled gratings and Ge substrate is used to calculate the decoupling efficiency of the device [59]. The optical properties of Au and Ge used in the model were taken from the data by Johnson and Palik *et al.* [61], [62] and the refractive index of SiO_2_ is fixed at 1.45. Different from the simulation of the coupling efficiency and electric field distribution, to simulate the optical band structure of the coupling grating, in the *x* directions, Bloch boundary conditions were applied to simulate infinite grating periods. In the *z* direction, perfectly matched layer (PML) boundary conditions were used to absorb the light waves and the incident light source is plane wave [56].

### 2.2 Device fabrication

Devices were fabricated on an intrinsic Ge wafer with a bulk resistivity of 50 Ω cm. The Ge wafer was cleaned by ultrasonication in acetone, isopropyl alcohol (IPA), and deionized water for 15 min, respectively. First, use contact lithography to fabricate the large contact pads that supply power to the IDEs. Sequentially spin-coat a 1 µm thick layer of LOR10a followed by a 1.4 µm thick layer of AZ5214 photoresist. After exposure and development, a layer of 10 nm Ti and 190 nm Au was deposited by e-beam evaporation. Next, lift-off the pattern by soaking in a solution of acetone and IPA. All alignment marks used in subsequent processes are simultaneously fabricated in the aforementioned steps. Subsequently, the core components of the device were prepared using two-step electron beam lithography (EBL, Pioneer Two 20 keV) combined with lift-off process. The first step is to make the bottom metal layer, waveguide and IDEs. First, spin-coat a 255 nm thick layer of PMMA A4 photoresist, followed by exposure with an exposure dose of 160 μC/cm^2^, and then developed for 100 s in a 3:1 solution of methyl isobutyl ketone (MIKA)/IPA and for 30 s in IPA alone. In the first step, a layer of 5 nm Ti and 80 nm Au was deposited by e-beam evaporation followed by lift-off process (Figure S8i). The process for fabricating the top coupling gratings in the second step is essentially the same as the first step, with the only difference being the thickness of the Au is 110 nm (Figure S8ii). Finally, approximately 800 nm of SiO_2_ was grown over the device using Plasma-Enhanced Chemical Vapor Deposition (PECVD) (Figure S8iii) and then spin-coat a 3 µm thick layer of AZ6130 photoresist. After exposure and development, use Reactive Ion Etching (RIE, SF_6_: 5 sccm, CHF_3_: 30 sccm, Ar: 20 sccm) to etch away the SiO_2_ on the contact pads to form openings for subsequent wire bonding (Figure S8iv). The contact pads were led out by an ultrasonic aluminum wire welder for subsequent characterization.

### 2.3 Optoelectronic testing system

Figure S10 is a schematic diagram of the self-built optoelectronic measurement system. We use three continuous wave lasers with wavelengths of 980 nm, 1,064 nm and 1,310 nm as pump light sources. Two lenses and a pinhole are used to collimate and filter the incident beam, and an infrared microscope objective (Thorlabs, MY50X-825, ×50, NA = 0.42) is employed to focus the beam onto the sample surface. A set of a linear polarizer and a half-wave plate are utilized to adjust the polarization state of incident light. A variable beam expander is utilized to adjust the size of the light spot onto the sample. The reflected light by the sample can be monitored and imaged by an infrared CCD camera. The measurements of the current-voltage (I–V) and photocurrent were performed with a Keithley 2450 source meter.

## 3 Results and discussion

**Figure 1:**
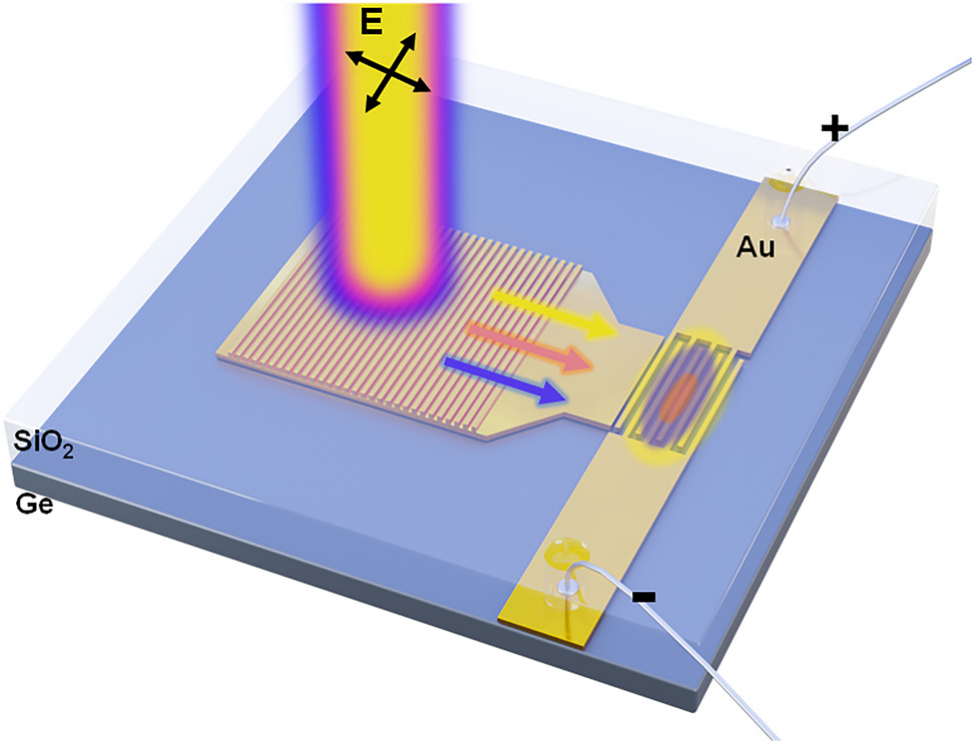
3D schematic diagram of the proposed plasmonic interconnect circuit.

Figure 1a shows the schematic diagram of the plasmonic interconnect circuit which we constructed. Similar to the previously reported structures [50], [58], the device consists of three core components: a surface plasmon coupler with Au subwavelength gratings, an Au-tapered waveguide, and an MSM photodetector with Au interdigitated electrodes (IDEs) for the excitation, propagation, and detection of optical field, respectively. The device is placed on the Ge substrate, therefore, in the near-infrared band, the device can have two types of photocurrent generation mechanisms: when bias is applied to the IDEs, electron-hole pairs are separated in the Ge substrate and collected by the Au IDEs, generating most of the photocurrent; Additionally, hot-electron generation and injection through the Au–Ge Schottky barrier also contribute to the photocurrent via internal photoemission (IPE). Unlike the single hot electron generation mechanism [63], [64], the photocurrent generation mechanism outlined above significantly enhances the responsiveness of the photocurrent. This improvement allows for a more precise differentiation of the variations in photocurrent that result from subtle changes in the optical modes.

**Figure 2:**
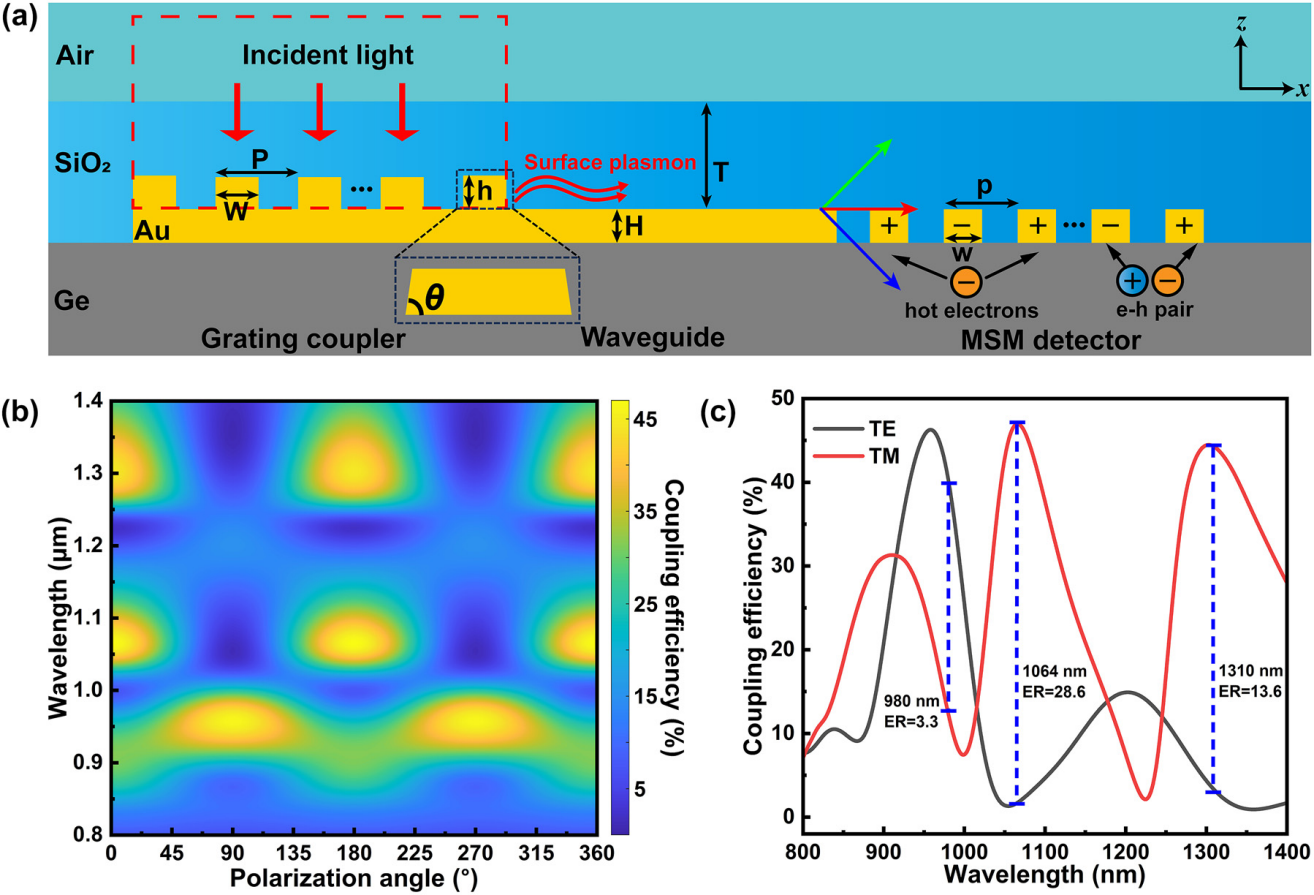
The working process and coupling efficiency of the proposed plasmonic interconnect circuit. (a) 2D structural diagram and working processes (see Supporting Information S1, Figure S3a) of the proposed plasmonic interconnect circuit with SiO_2_ refractive index matching layer in *x*–*z* plane. (b) Coupling efficiency as a function of polarization angle in the 800–1,400 nm wavelength range. (c) Coupling efficiency between TM and TE-polarization in Figure 2b (*P* = 907.6 nm, *W* = 420 nm, *h* = 115.6 nm, *θ* = 75° and *T* = 800 nm).

Figure 2a presents a detailed cross-sectional view of the plasmonic interconnect circuit schematic. The plasmonic interconnect circuit proposed in this paper has two significant structural improvements. Firstly, we coated the SiO_2_ refractive index matching layer on top of the device, according to Equation (1), the SPP coupling condition of the gratings can be expressed as [9]:

(1) $$k_{\text{SPP}}=k_{0}\sqrt{\frac{\varepsilon_{d}\cdot \varepsilon_{m}}{\varepsilon_{d}+\varepsilon_{m}}}=\frac{2\pi }{\lambda_{0}}\cdot n_{i}\cdot \sin\theta +m\cdot \frac{2\pi }{P}$$

where *k*_SPP_ is the surface plasmon wave vector, *k*_0_ is the wave vector of the light in vacuum, *P* is the grating period, *m* is the diffraction order, *λ*_0_ is the wavelength of the incident light in vacuum, *n*_*i*_ is the refractive index of the medium, *ε*_*d*_ and *ε*_*m*_ are the dielectric constants of the medium and the metal, respectively, and *θ* is the angle of incident light. It is obvious that when the medium changes from air (*n* = 1) to SiO_2_ (*n* ≈ 1.45), the corresponding *P* can decrease. So, this approach can, to a certain extent, reduce the size of the grating coupler, which is advantageous for enhancing the device’s integration [59]. Although actual grating couplers often use multiple grating periods to accommodate the size of the incident light spot, leading to a larger overall size, previous simulations indicate that a configuration of approximately seven grating periods can provide effective coupling and the size of such a configuration is nearly on par with the diffraction limit of the focused light spot [58]. Beyond the potential advantage of reducing the footprint size of the device, the most crucial role of incorporating the refractive index matching layer is tantamount to establishing a dielectric waveguide atop the plasmonic structures, this configuration yields numerous optical modes that are highly sensitive to the excitation wavelength and polarization state, which will be discussed in detail in the subsequent sections. In addition to the refractive index matching layer, another key structural improvement is focused on the grating coupler. Within the context of optical mode detection in this paper, the grating coupler must meet certain requirements: Firstly, it should have high coupling efficiency to ensure that sufficient optical field energy is transmitted and then converted into photocurrent. Secondly, the optical modes generated when subjected to different incident light states must be distinct enough to be effectively distinguished and detected. Therefore, we used the two-dimensional (2D) finite-difference time domain (FDTD) method combined with the PSO algorithm to optimize the design of the grating coupler. As shown in the Figure 2a, we optimized the five structural parameters of the coupler, which are: the period (*P*) and duty cycle (*W*/*P*) of the coupling grating, the height (*h*) and tilt angle (*θ*) of the top grating, and the thickness (*T*) of the refractive index matching layer.

It should be noted that we adopted the design of top grating on the waveguide. This composite structure, similar to the previously reported stepped metal grating coupler [25], offers two primary advantages over gratings that are directly embedded on substrates. Firstly, the bottom waveguide acts as a metal reflector, which greatly improves the coupling efficiency of the coupler [59]. Secondly, the bottom metal layer serves as a barrier, preventing light leakage into the substrate beneath the coupler [51]. Consequently, the pure photocurrent detected in the MSM photodetector primarily originates from the optical field above the waveguide. This feature is vital for precisely differentiating between various optical modes by analyzing the variations in photocurrent. The bottom metal waveguide layer can be fabricated simultaneously with the IDEs, so they share the identical height (*H*). This height needs to ensure that it can fully block light from entering the substrate below the grating coupler, while also not affecting the scattering of the light field at the waveguide end into the substrate below the IDEs to efficiently generate photocurrent. Based on previous design experience, we have set the height to 80 nm [59]. In addition, the MSM structure has not undergone specific optimization. Previous research indicates that smaller grating periods and duty cycles lead to higher decoupling efficiency [50]. However, considering the practical manufacturing constraints, we selected structural parameters (The period *p* of the decoupling gratings is 800 nm with a duty cycle of 50 %) that are relatively easy to fabricate.

Our primary optimization objective for the five structural parameters of the grating coupler is to attain maximum coupling efficiency at 1,310 nm and TM-polarization. The initial optimization results indicate that with top layer grating period, duty cycle, height, tilt angle, and SiO_2_ layer thickness set at 907.6 nm, 46.22 % (*W* ≈ 420 nm), 115.6 nm, 75° and 868.5 nm, the coupling efficiency can achieve approximately 46 % at 1,310 nm (Supporting Information S1, Figure S1). Additionally, the coupler features multiple peaks of coupling efficiency across various wavelength bands and distinct polarization states (Supporting Information S1, Figure S2). Conversely, comparable structural device lacking refractive index matching layer exhibit only a single peak of coupling efficiency in the designated band and TM-polarization, which indicates that the grating coupler with refractive index matching layer can support multiple optical modes (Supporting Information S1, Figure S3).

To facilitate the identification and detection of these optical modes, it is crucial that the coupler should exhibit significant differences in coupling efficiency across various polarization states and wavelength. Moreover, we aim for the different peaks of coupling efficiency to coincide with the wavelengths of commonly used lasers, facilitating experimental detection and future practical applications. To achieve this goal, it is usually necessary to redesign the parameters of the coupler gratings, especially the period. In our previous work, we reported a more convenient method: adjusting the refractive index of the matching layer to achieve tunable grating couplers [59]. In this work, the introduction of various optical modes by the refractive index matching layer makes these modes sensitive not only to the refractive index but also to the layer’s thickness. Consequently, we seek to modulate the peak position of coupling efficiency by varying the thickness of the refractive index matching layer. Maintaining all other parameters constant, we conducted a scan of the coupling efficiency across various thicknesses of the refractive index matching layer (Supporting Information S2, Figure S4d). As the thickness (*T*) decreases from 868.5 nm to 800 nm, the grating coupler exhibits distinct polarization sensitivity difference in the vicinity of the 980 nm, 1,064 nm, and 1,310 nm wavelength bands (Figure 2b). Figure 2c shows the comparison of coupling efficiency of the further optimized grating coupler under TM and TE-polarization state. The grating coupler can achieve the coupling efficiency of about 45 % at wavelengths of 1,310 nm, 1,064 nm and 980 nm. Importantly, the polarization dependence of the coupling efficiency at 980 nm is completely inverse compared at 1,310 nm and 1,064 nm. In addition, there is also a certain difference in the polarization contrast of coupling efficiency at the aforementioned wavelengths, with extinction ratios (*ER*) reaching 28.6 and 13.6 at 1,064 nm and 1,310 nm (TM/TE), respectively, while only 3.3 at 980 nm (TE/TM). The difference in coupling efficiency under different polarization states will directly affect the contrast of the subsequently generated photocurrent, and is expected to become a key indicator for electrical detection and differentiation of different optical modes.

**Figure 3:**
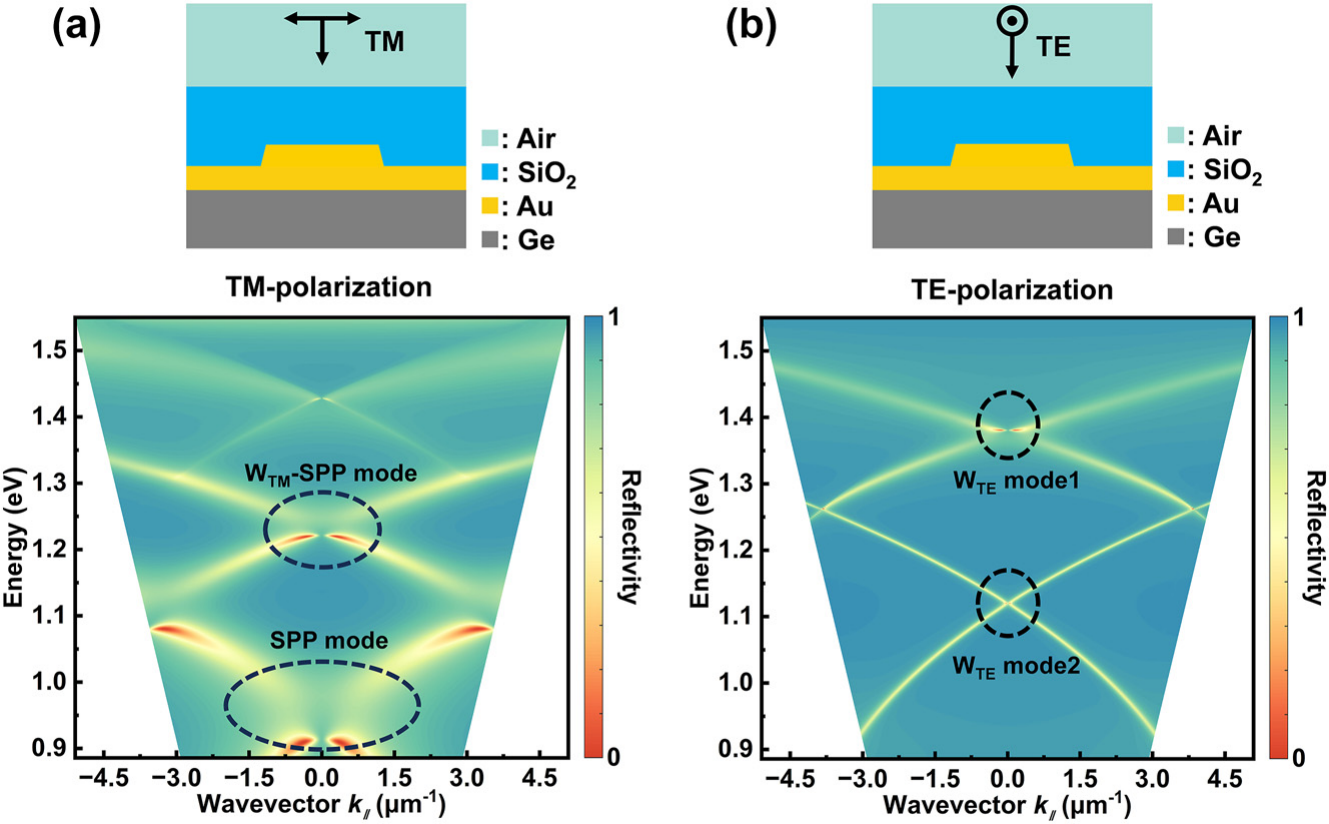
Simulated optical band structure of the coupling grating under (a) TM and (b) TE incident light. Band structure in energy (*E*) − wavevector (*k*_//_) format was plotted by converting the wavelength (*λ*) − incident angle (*θ*) relation to the *E*−*k*_//_ relation based on *E* = *hc*/*λ* and *k*_//_ = (2*π*/*λ*) sin*θ*.

To reveal the optical modes behind the formation of the different coupling efficiency peaks mentioned above, Figure 3 shows the simulated angular-resolved reflectance spectrum which reflects optical band structure of the coupling grating under TM and TE-polarization. It is evident that at different photon energy, whether illuminated by TM or TE-polarization light, the optical band of the grating structure presents multi branch dispersion topography, which is consistent one-to-one with the multiple peaks of the coupling efficiency as shown in Figure 2c. The optical band structure encompasses a variety of optical modes, including pure SPP mode, waveguide mode characterized by field concentration within the refractive index matching layer, and hybrid coupling mode that represent an interaction between SPP and waveguide mode. On the contrary, in the grating structure without SiO_2_ refractive index matching layer coverage, the optical band structure only exhibits a single pure SPP mode under TM-polarization (Supporting Information S3, Figure S6). Correspondingly, Figure S7 shows the coupling efficiency of the device under different incident angles of illumination. It can be seen that at the TM and TE-polarization states, the coupling efficiency peaks corresponding to each optical mode exhibit a relatively coordinated drift, each mode can still exist stably. In addition, due to the asymmetric propagation of the light field caused by oblique incidence, the coupling efficiency corresponding to each optical mode can exceed 60 %. Indeed, oblique incidence is an effective means to improve the coupling efficiency of grating couplers. However, our current testing system is unable to achieve precise oblique incidence control, so the structure in this article is designed based on normal-incidence beam, and with certain parameter adjustments, the device can fully operate in the oblique incidence state.

**Figure 4:**
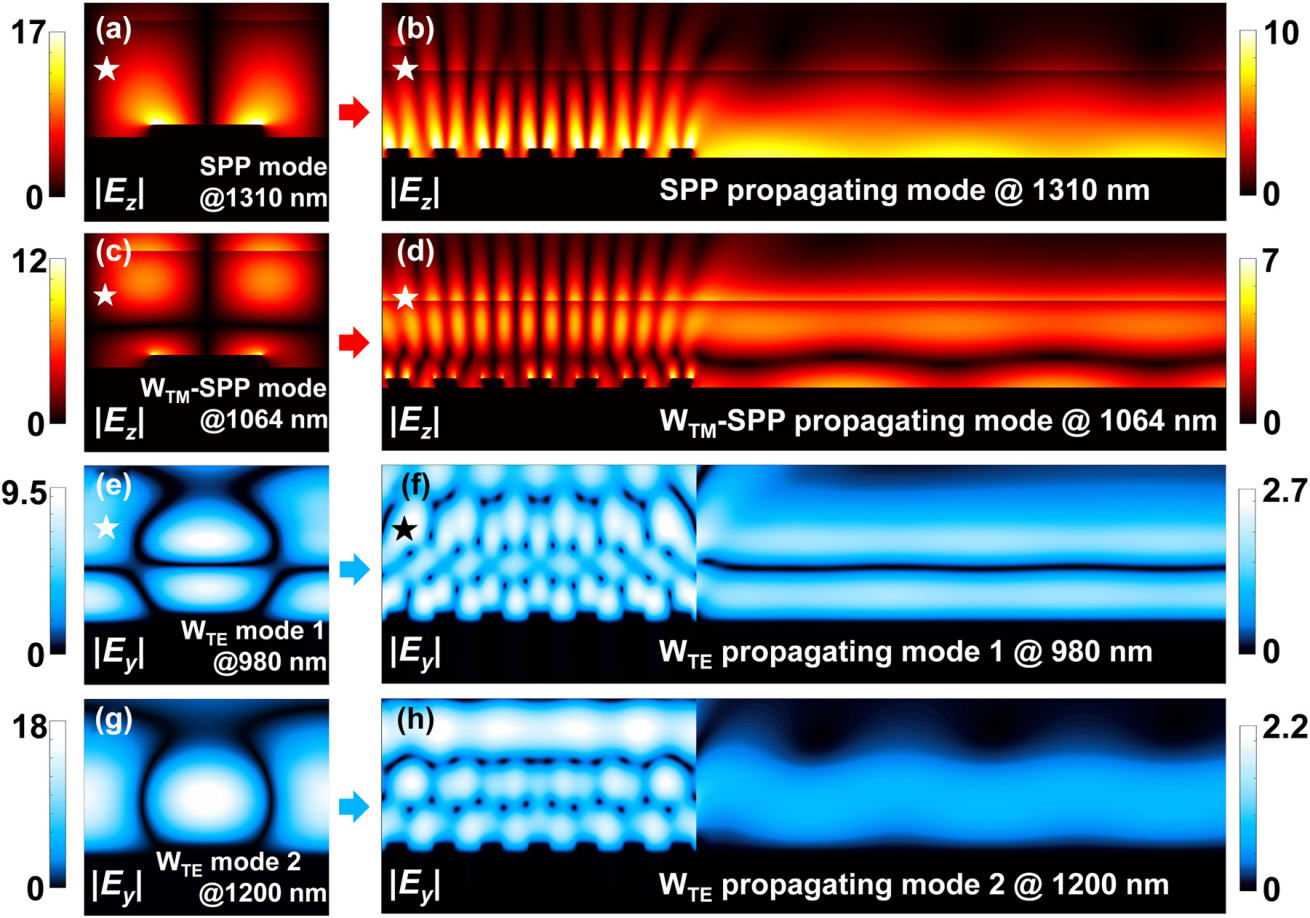
Simulated optical near fields and propagating light fields of the plasmonic interconnect circuit at different wavelengths and polarization states. (a, c, e, g), respectively, represent the optical near-field distribution with pure SPP, W_TM_-SPP and W_TE_ mode. (b, d, f, h) are the corresponding propagating modes in (a, c, e, g). The pentagram symbol represents that this optical mode can achieve effective propagation.

Figure 4 visually illustrates the near-field origin and propagation evolution of various optical modes. On the left side of the figure is the simulated electric field distribution of a single grating periodic unit, the red color represents the |*E*_*z*_| component under TM-polarization, while blue color represents the |*E*_*y*_| component under TE-polarization. On the right is the corresponding electric field distribution of the grating coupler with 7 grating periods under total-field scatted-field (TFSF) incident light source, as well as the propagation field distribution on the subsequent Au/SiO_2_ waveguide interface. Figure 4a and b shows typical and pure SPP near-field and propagating mode at 1,310 nm, the electric field is localized at the tip of the gratings and propagates closely against the surface of the Au waveguide [50], [58], [59]. Similarly, Figure 4c and d clearly demonstrate the hybrid TM waveguide-SPP (W_TM_-SPP) mode at 1,064 nm. Since TE-polarized light cannot excite SPP, Figure 4e–h exclusively exhibit pure TE waveguide (W_TE_) modes at 980 nm and 1,200 nm. The light field at 1,200 nm is firmly confined in the dielectric layer with a very high-quality factor which can be represented by the extremely narrow band in Figure 3b [65]. Therefore, W_TE_ mode 2 cannot achieve effective propagation as shown in Figure 4g and h which corresponds to the low coupling efficiency in this wavelength compared to the other peaks in Figure 2c. The above propagating light modes can be approximated as the conformal continuation of the optical near-field in spatial distribution, thereby creating efficient links between localized optical near-fields and propagating light fields.

**Figure 5:**
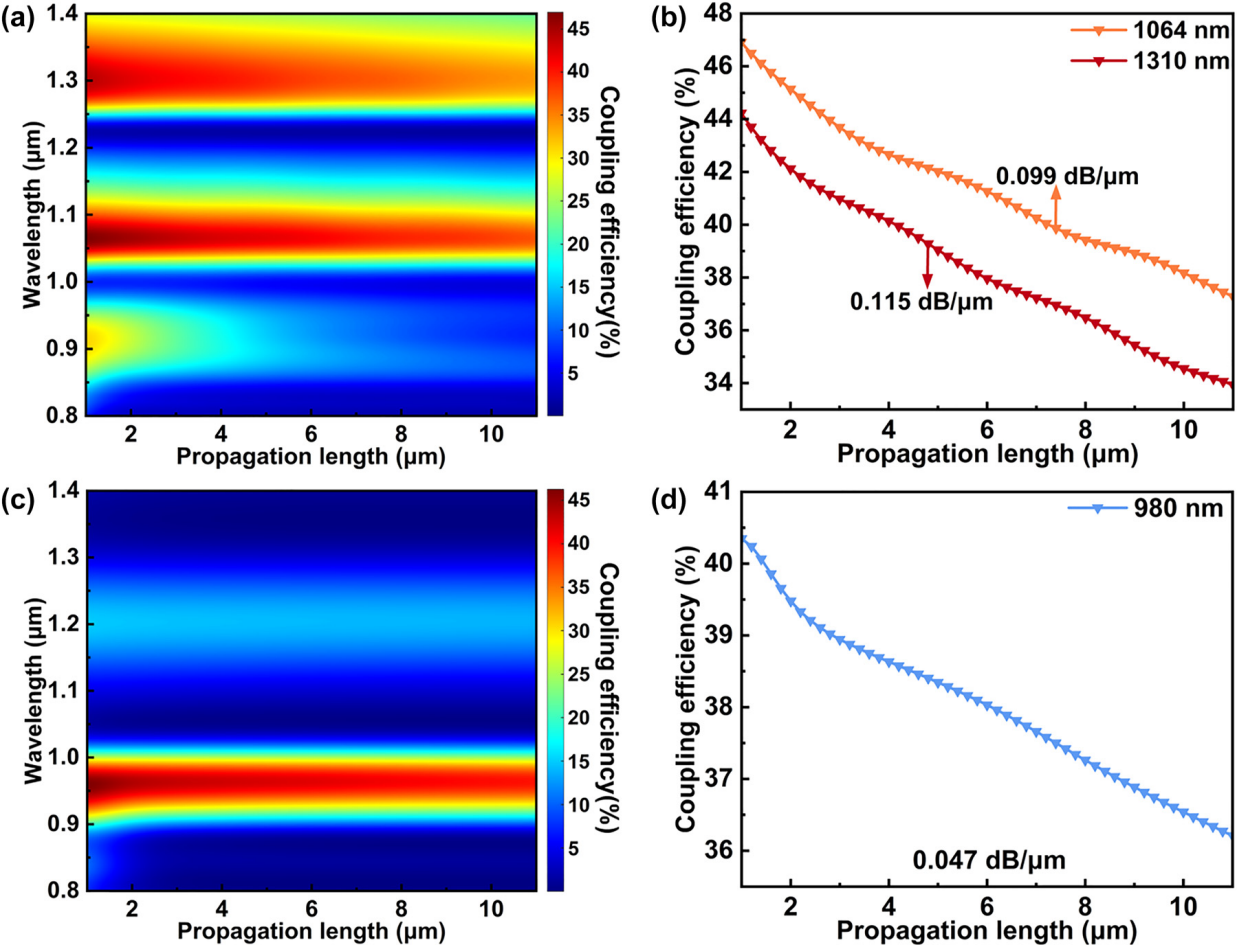
Coupling efficiency as a function of different propagation length in the 800–1,400 nm wavelength range at (a) TM and (c) TE-polarization, (b) the relationship between coupling efficiency and propagation length at 1,310 nm and 1,064 nm at TM-polarization. (d) The relationship between coupling efficiency and propagation length at 980 nm at TE-polarization.

Figure 5a and c shows the coupling efficiency of the device with different propagation length at TM and TE-polarization. We use the coupling efficiency at a distance of 1 μm from the end of the coupling gratings as the reference power point (*P*_0_), and the coupling efficiency at a distance of 11 μm as the detection power point (*P*_1_). The mode propagation loss can be expressed as: −10log(*P*_1_/*P*_0_), The loss coefficient (*α*) is the propagation loss divided by the propagation length (*L*). As shown in Figure 5b and d, the loss coefficient of the SPP mode (1,310 nm) and W_TM_-SPP mode (1,064 nm) under TM-polarization is 0.115 dB/μm and 0.099 dB/μm, respectively, and the loss coefficient of the W_TE_ mode 1 (980 nm) is 0.047 dB/μm. It can be seen that the optical mode with spatial distribution shifted towards the medium has a smaller loss coefficient, this is precisely the design concept of the hybrid plasmonic mode [40].

**Figure 6:**
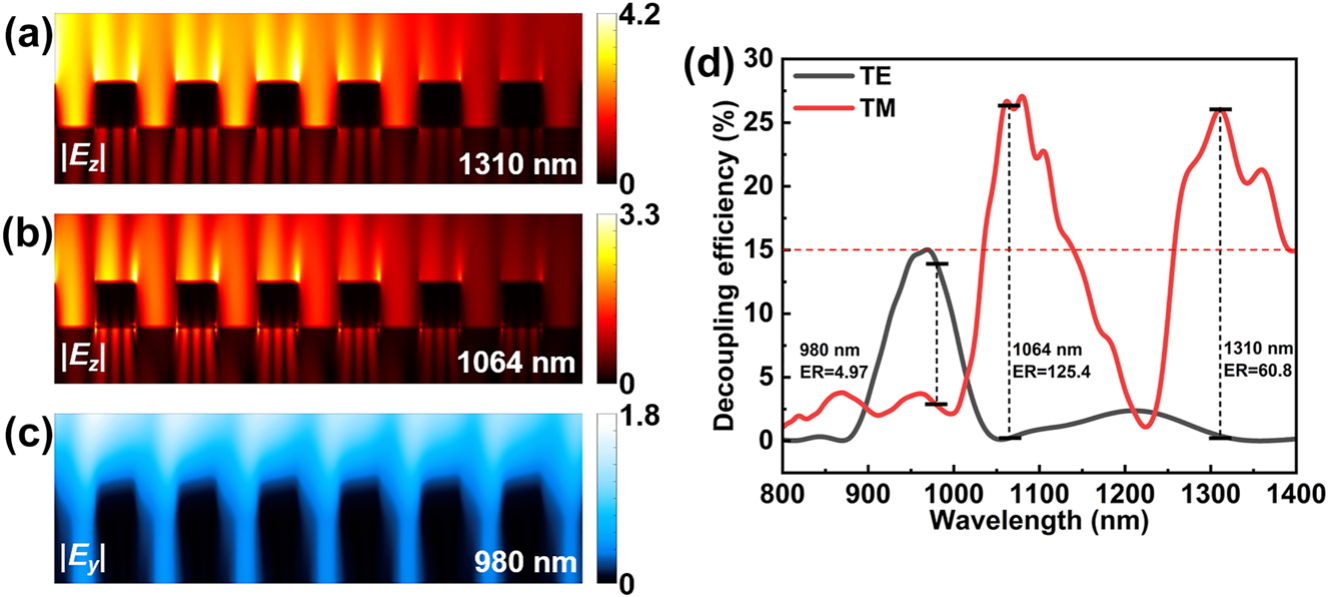
Working characteristics of the decoupling gratings. (a–c) The electric field distribution at the decoupling gratings when the device transmits different optical modes, (a) SPP mode (1,310 nm, TM-polarization), (b) W_TM_-SPP mode (1,064 nm, TM-polarization), (c) W_TE_ mode (980 nm, TE-polarization). (d) Decoupling efficiency between TM and TE-polarization.

Figure 6a–c shows the electric field distribution at the decoupling gratings when the device transmits different optical modes, the light field can be effectively decoupled into the substrate. The trend of decoupling efficiency is basically consistent with the coupling efficiency as shown in Figure 6d, this synergy benefits from the high coupling efficiency of the device, which provides sufficient energy to reach the photodetection component and compensate for the sensitivity differences of the decoupled gratings to different wavelengths and optical modes [59]. In addition, Figure S5 shows the variation of device decoupling efficiency with SiO_2_ layer thickness, which is highly consistent with the variation of coupling efficiency with *T*, this further proves that adjusting the peak position of optical modes by changing the thickness of the SiO_2_ layer is effective.

**Figure 7:**
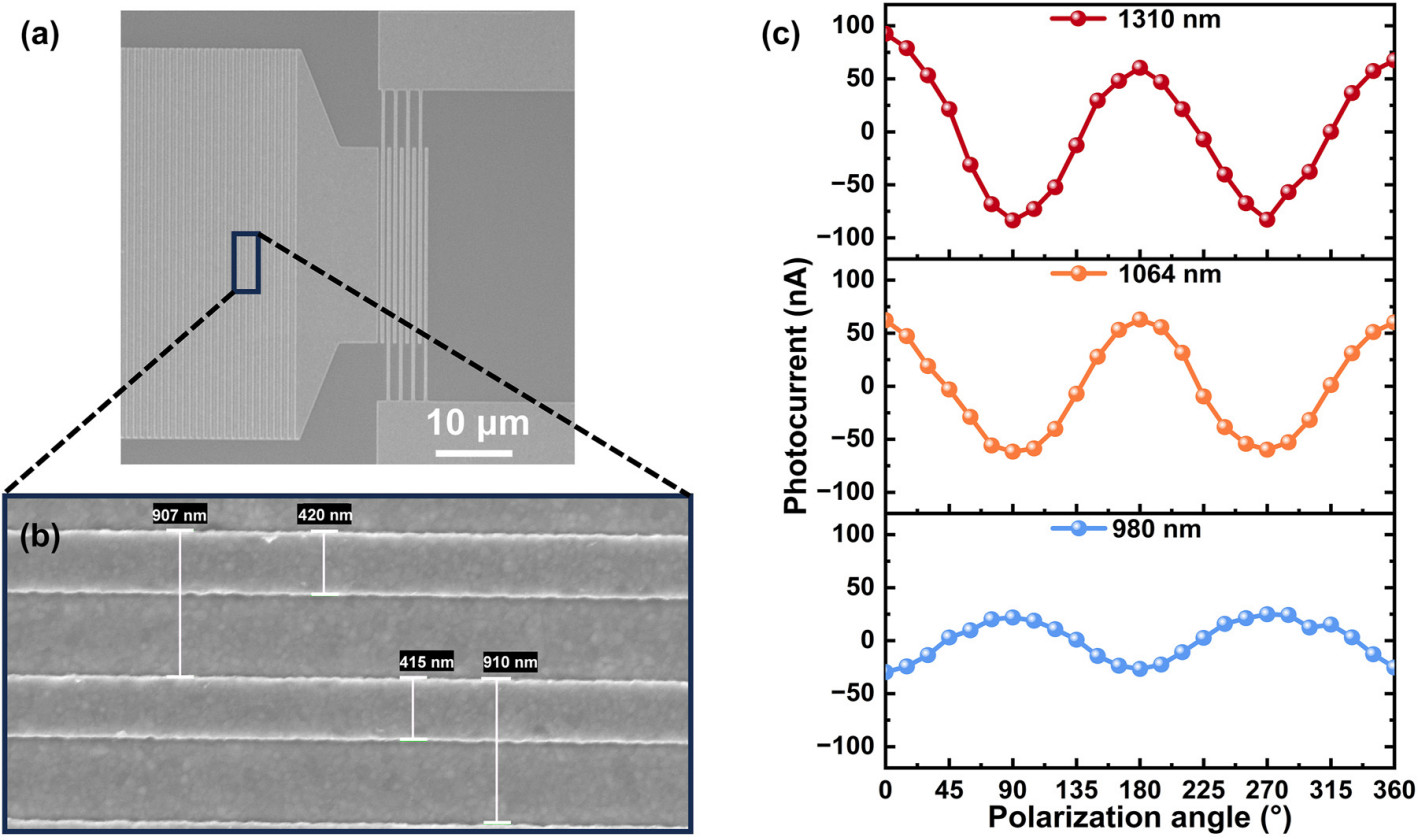
Actual preparation and optoelectronic performance of the plasmonic interconnect circuit. (a) SEM image of the fabricated plasmonic interconnect circuit. (b) Local magnification in (a). (c) Photocurrent as a function of polarization angle for different wavelengths (mean value of the photocurrent is subtracted).

We actually fabricated the designed plasmonic interconnect circuit as shown in Figure 7a (Supporting Information S4, Figures S8 and 9). Figure 7b shows a locally magnified SEM image of the coupled gratings, demonstrating relatively good process accuracy. We tested the photocurrent response of the plasmonic interconnect circuit at different polarization states and wavelengths of 980 nm, 1,064 nm and 1,310 nm using the self-built optoelectronic measurement system (Supporting Information S5, Figure S10). The dark I–V characteristic of the MSM photodetector is shown in Figure S11, the detector consists of two identical Schottky diodes connected back-to-back, which shows similar behavior under both positive and negative bias [50], [58]. In the actual optoelectronic testing in Figure 7c, the diameter of the incident focusing spot is about 42 μm, the optical power is fixed at 200 μW, and a bias voltage of −0.03 V is applied to the IDEs.

In addition, to explore the basic performance of MSM photodetector, we tested its responsivity and response speed at 1,310 nm and TM-polarization (Supporting Information S6, Figures S11 and 12). Figure S12a shows the responsivity, mainly due to the large transmission loss, the responsivity (≈1.24 mA/W@1,310 nm) of photodetectors is low, and the external quantum efficiency (EQE ≈ 0.173 %) is also small. However, the device demonstrated a relatively fast response time (≈537 ns, as shown in Figure S12b). In short, the photodetector in this article is only functional and its performance is not outstanding. We can use two-dimensional materials such as graphene laid flat on waveguides to achieve efficient optoelectronic detection [33]. However, this undoubtedly increases the complexity and cost of the process.

It should be pointed out that compared with our previous work [58], the structure optimized in this article has improved the coupling efficiency, but the obtained photocurrent seems to be inferior to the original structure, mainly due to the following two reasons: ① The waveguide length of the device in this paper is 10 μm, which is twice as long as our previous work (5 μm). This will result in significant transmission losses.② The area of the photodetector in this paper is about half the size of that in our previous work. This will also severely reduce the photocurrent collection efficiency of the devices.

In the above three wavelengths, the photocurrent exhibits strong polarization correlation, while the polarization correlation at 980 nm is completely opposite to that at 1,064 nm and 1,310 nm. In addition, the polarization contrast of the photocurrent at 1,064 nm and 1,310 nm is significantly greater than that at 980 nm, which is consistent with the simulated coupling and decoupling efficiency characteristics. This indicates that it is feasible to use electrical methods to detect and identify optical modes in our designed plasmonic interconnect circuit. Figure S13 shows the polarization extinction ratio (TM/TE) of the decoupling efficiency at different SiO_2_ thicknesses. It must be explained that the polarization extinction ratio of the photocurrent at 1,064 nm is lower than predicted, as the high extinction ratio region for this mode is narrow (as shown in Figure S13a and b). Process errors (especially the thickness of SiO_2_) can cause shifts in the coupling and decoupling efficiency spectral line, leading to significant degradation of the extinction ratio at 1,064 nm. It can be seen that precise performance quantification analysis requires at least improvements in device fabrication accuracy and enhancements in photodetector performance. This work currently mainly demonstrates a new working form of the plasmonic interconnect circuit, develops an appropriate research platform, and experimentally verifies its feasibility. The optimization direction and working mode of this device in the future can be as follows: ① Minimize process errors and employ higher-performing photoelectric conversion structures to achieve photocurrents that better align with simulation expectations. ② Test the photocurrent of devices under different optical modes, not only in terms of magnitude, but also in different polarization states and wavelengths, forming a database (calibrated photocurrent). ③ Compare the real-time status of the device’s photocurrent with the calibrated photocurrent. When a discrepancy is detected, it indicates that the device is not operating in the designated optimal optical mode. The degree of deviation can be quantified based on numerical differences such as the polarization extinction ratio.

## 4 Conclusions

In summary, we have carefully designed a plasmonic interconnect circuit featuring the integration of a subwavelength metal grating coupler and an MSM photodetector linked through a metal waveguide. Utilizing particle swarm optimization, we fine-tuned the coupler’s structural parameters to enhance signal transmission efficiency and mode differentiation at crucial near-infrared wavelengths. Thanks to the precise control of optical modes in the device by the refractive index matching layer, our design refinement facilitated selective excitation and efficient detection of various optical modes, underscoring the device’s potential in precise optical signal manipulation. The experimental results, aligned with simulation predictions, confirmed the effectiveness in on chip electrical detecting distinct optical modes which links between localized optical near fields and propagating light fields. This efficient linking is crucial for various applications in nanophotonic and plasmonic, as it enables the transfer of information and energy between different scales and formats of light. Our work aims to establish a new way of connection between electronic circuits and photonic circuits, contributing to the future applications in sensing, signal processing, and communication.

## Supplementary Material

Supplementary Material Details

## References

[1] Ozbay E. (2006). Plasmonics: merging photonics and electronics at nanoscale dimensions. *Science*.

[2] Brongersma M. L., Shalaev V. M. (2010). Applied physics the case for plasmonics. *Science*.

[3] Zhang W. P. (2024). A system-on-chip microwave photonic processor solves dynamic RF interference in real time with picosecond latency. *Light Sci. Appl.*.

[4] Reed G. T., Mashanovich G., Gardes F. Y., Thomson D. J. (2010). Silicon optical modulators. *Nat. Photonics*.

[5] Shu H. W. (2022). Microcomb-driven silicon photonic systems. *Nature*.

[6] Feng H. (2024). Integrated lithium niobate microwave photonic processing engine. *Nature*.

[7] Xiang C. (2023). 3D integration enables ultralow-noise isolator-free lasers in silicon photonics. *Nature*.

[8] Zia R., Schuller J. A., Chandran A., Brongersma M. L. (2006). Plasmonics: the next chip-scale technology. *Mater. Today*.

[9] Dragoman M., Dragoman D. (2008). Plasmonics: applications to nanoscale terahertz and optical devices. *Prog. Quant. Electron.*.

[10] Ebbesen T. W., Genet C., Bozhevolnyi S. I. (2008). Surface-plasmon circuitry. *Phys. Today*.

[11] Sorger V. J., Oulton R. F., Ma R. M., Zhang X. (2012). Toward integrated plasmonic circuits. *MRS Bull.*.

[12] Wu H. (2022). Photonic nanolaser with extreme optical field confinement. *Phys. Rev. Lett.*.

[13] Wang Y. (2020). Stable, high-performance sodium-based plasmonic devices in the near infrared. *Nature*.

[14] Fernandez-Bravo A. (2019). Ultralow-threshold, continuous-wave upconverting lasing from subwavelength plasmons. *Nat. Mater.*.

[15] Wu H. (2019). Plasmonic nanolasers: pursuing extreme lasing conditions on nanoscale. *Adv. Opt. Mater.*.

[16] Li C., Liu Z., Shang Q. Y., Zhang Q. (2019). Surface-plasmon-assisted metal halide perovskite small lasers. *Adv. Opt. Mater.*.

[17] Du W., Wang T., Chu H. S., Nijhuis C. A. (2017). Highly efficient on-chip direct electronic-plasmonic transducers. *Nat. Photonics*.

[18] Wang Z. (2024). Upconversion electroluminescence in 2D semiconductors integrated with plasmonic tunnel junctions. *Nat. Nanotechnol.*.

[19] Doderer M. (2023). Broadband tunable infrared light emission from metal-oxide-semiconductor tunnel junctions in silicon photonics. *Nano Lett.*.

[20] Chelladurai D., Doderer M., Koch U., Fedoryshyn Y., Haffner C., Leuthold J. (2019). Low-loss hybrid plasmonic coupler. *Opt. Express*.

[21] Fang Y. R., Sun M. T. (2015). Nanoplasmonic waveguides: towards applications in integrated nanophotonic circuits,” (in English). *Light Sci. Appl.*.

[22] Yang L. (2023). Generating a sub-nanometer-confined optical field in a nanoslit waveguiding mode. *Adv. Photonics*.

[23] Wei H. (2018). Plasmon waveguiding in nanowires. *Chem. Rev.*.

[24] Messner A., Moor D., Chelladurai D., Svoboda R., Smajic J., Leuthold J. (2023). Plasmonic, photonic, or hybrid? Reviewing waveguide geometries for electro-optic modulators. *APL Photonics*.

[25] Ayata M. (2017). High-speed plasmonic modulator in a single metal layer. *Science*.

[26] Messner A. (2021). Broadband metallic fiber-to-chip couplers and a low-complexity integrated plasmonic platform. *Nano Lett.*.

[27] Haffner C. (2018). Low-loss plasmon-assisted electro-optic modulator. *Nature*.

[28] Kohli M. (2023). Plasmonic ferroelectric modulator monolithically integrated on SiN for 216 GBd data transmission. *J. Lightwave Technol.*.

[29] Thomaschewski M., Zenin V. A., Fiedler S., Wolff C., Bozhevolnyi S. I. (2022). Plasmonic lithium niobate Mach–Zehnder modulators. *Nano Lett.*.

[30] Yezekyan T., Thomaschewski M., Thrane P. C. V., Bozhevolnyi S. I. (2024). Plasmonic electro-optic modulators on lead zirconate titanate platform. *Nanophotonics*.

[31] Koepfli S. M. (2023). Metamaterial graphene photodetector with bandwidth exceeding 500 gigahertz. *Science*.

[32] Salamin Y. (2018). 100 GHz plasmonic photodetector. *ACS Photonics*.

[33] Ma P. (2019). Plasmonically enhanced graphene photodetector featuring 100 Gbit/s data reception, high responsivity, and compact size. *ACS Photonics*.

[34] Dorodnyy A. (2018). Plasmonic photodetectors. *IEEE J. Sel. Top. Quantum Electron.*.

[35] Guo J. S. (2020). High-performance silicon-graphene hybrid plasmonic waveguide photodetectors beyond 1.55 μm. *Light Sci. Appl.*.

[36] Jian J. L. (2023). High-speed compact plasmonic-PdSe_2_ waveguide-integrated photodetector. *ACS Photonics*.

[37] Wang Y. L. (2021). Ultra-compact high-speed polarization division multiplexing optical receiving chip enabled by graphene-on-plasmonic slot waveguide photodetectors. *Adv. Opt. Mater.*.

[38] Thraskias C. A. (2018). Survey of photonic and plasmonic interconnect technologies for intra-datacenter and high-performance computing communications. *IEEE Commun. Surv. Tutorials*.

[39] Liu Y. (2020). The design of CMOS-compatible plasmonic waveguides for intra-chip communication. *IEEE Photonics J.*.

[40] Sun P. F., Xu P. F., Zhu K. J., Zhou Z. P. (2021). Silicon-based optoelectronics enhanced by hybrid plasmon polaritons: bridging dielectric photonics and nanoplasmonics. *Photonics*.

[41] Hoessbacher C. (2021). Progress and challenges of plasmonics for efficient and high-speed optical communications. *Conference on Lasers and Electro-Optics (CLEO), Electr Network, May 09–14 2021, NEW YORK: Ieee, in Conference on Lasers and Electro-Optics*.

[42] Leuthold J. (2021). Plasmonic data center interconnects (DCIs). *Optical Fiber Communications Conference and Exhibition (OFC), Electr Network, Jun 06–11 2021*.

[43] Koch U. (2020). A monolithic bipolar CMOS electronic-plasmonic high-speed transmitter. *Nat. Electron.*.

[44] Fukuda M., Tonooka Y., Inoue T., Ota M. (2019). Feasibility of plasmonic circuits for on-chip interconnects. *Solid-State Electron.*.

[45] Fukuda M., Okahisa S., Tonooka Y., Ota M., Aihara T., Ishikawa Y. Feasibility of plasmonic circuits in nanophotonics. *IEEE Access*.

[46] Noor S. L., Catthoor F., Lin D. N., Van Dorpe P., Naeemi A. (2024). Comparison of photonic to plasmonic mode converters for plasmonic multiple-input devices. *IEEE Photonics J*..

[47] Meng Y. (2021). Optical meta-waveguides for integrated photonics and beyond. *Light Sci. Appl.*.

[48] Xu Q. (2023). Meta-optics inspired surface plasmon devices. *Photonics Insights*.

[49] Ji J. T., Zhai Y. S., Wu Z. P., Ma X. Y., Wang Q. L. (2020). Wavelength-polarization multiplexer for routing and detection of surface plasmon polaritons based on plasmonic gratings. *ACS Photonics*.

[50] Panchenko E. (2018). In-plane detection of guided surface plasmons for high-speed optoelectronic integrated circuits. *Adv. Mater. Technol.*.

[51] He X. B. (2022). On-chip detection of multiwavelength surface plasmon polaritons based on plasmonic demultiplexers. *ACS Photonics*.

[52] Nikolajsen T., Leosson K., Bozhevolnyi S. I. (2004). Surface plasmon polariton based modulators and switches operating at telecom wavelengths. *Appl. Phys. Lett.*.

[53] Temnov V. V. (2010). Active magneto-plasmonics in hybrid metal-ferromagnet structures. *Nat. Photonics*.

[54] Liu Y., Zhang J. S., Peng L. M. (2018). Three-dimensional integration of plasmonics and nanoelectronics. *Nat. Electron.*.

[55] Guo R. (2017). High-bit rate ultra-compact light routing with mode-selective on-chip nanoantennas. *Sci. Adv.*.

[56] Guan J. (2020). Quantum dot-plasmon lasing with controlled polarization patterns. *ACS Nano*.

[57] Park J. E. (2022). Polariton dynamics in two-dimensional ruddlesden-popper perovskites strongly coupled with plasmonic lattices. *ACS Nano*.

[58] Zhang C. R., Ma X. Y., Zhai Y. S., Wu Z. P., Xu Y. J., Wang Q. L. (2022). Unidirectional coupling and efficient detection of near-infrared surface plasmon polaritons for on-chip optoelectronic interconnection. *Opt. Express*.

[59] Zhang C. R., Wu Z. P., Wang Q. L. (2022). Tunable and efficient near-infrared plasmonic interconnect circuit based on an index matching layer and a metal reflector. *Opt. Mater. Express*.

[60] Jin J. J. (2019). Polarization-controlled unidirectional excitation of surface plasmon polaritons utilizing catenary apertures. *Nanoscale*.

[61] Johnson P. B., Christy R. W. (1972). Optical constants of noble metals. *Phys. Rev. B*.

[62] Palik E. D. (1997). Chapter 2 – refractive index. *Handbook of Optical Constants of Solids*.

[63] Fukuhara M., Ota M., Sakai H., Aihara T., Ishii Y., Fukuda M. (2014). Low-loss waveguiding and detecting structure for surface plasmon polaritons. *Appl. Phys. Lett.*.

[64] Sakai H. (2016). Plasmonic and electronic device-based integrated circuits and their characteristics. *Solid-State Electron.*.

[65] Hu P. (2022). Global phase diagram of bound states in the continuum. *Optica*.

