# Supplementary material for: On chip control and detection of complex SPP and waveguide modes based on plasmonic interconnect circuits
Source: Nanophotonics. 2024 Sep 9;13(23):4243–54. doi: 10.1515/nanoph-2024-0298 (PMC11636411; doi:10.1515/nanoph-2024-0298)
Supplement: Supplementary file 1 — Supplementary Material Details [file j_nanoph-2024-0298_suppl_001.docx]

Supplementary Materials for

**On chip control and detection of complex SPP and waveguide modes based on plasmonic interconnect circuits**

Canran Zhang *et al.*

*Corresponding author. Email: northrockwql@seu.edu.cn

**S1. Optimization process** **of coupling efficiency; Comparison with the device without refractive index matching layer.**

We use the FDTD solution's built-in optimization module to optimize the coupling efficiency based on PSO algorithm. The optimization direction is to achieve the highest possible coupling efficiency at 1310 nm. Among the 5 optimization parameters mentioned in the main text, the theoretical reference value of *P* can be obtained based on Equation (1), while the other parameters can been set with approximate value ranges based on past design experience and actual processing technology limitations. After 30 generations of optimization with 11 members, the coupling efficiency increased to 46.5% as shown in Figure. S1.


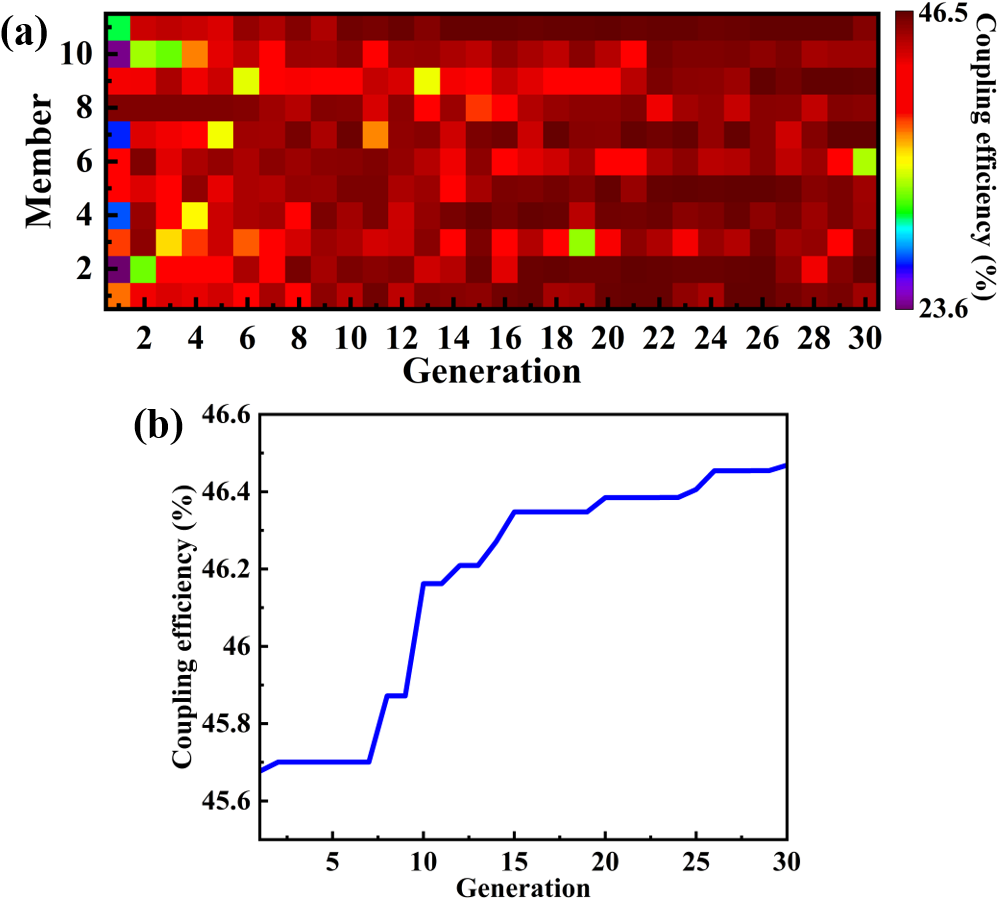


**Figure. S1.** (**a**) Optimization process of coupling efficiency. (**b**) The optimal value for each generation.

Despite the above optimized design, the grating coupler can achieve extremely high coupling efficiency at 1310 nm, however, the other occurrence wavelengths of the coupling efficiency peaks are unplanned (Figure. S2). We hope to achieve coupling efficiency peaks at several commonly used laser wavelengths such as 1064 nm and 980 nm, and to maximize the difference between TM and TE-polarization for practical testing and differentiation. To move the peak position of coupling efficiency, it is usually necessary to change the period of the gratings, but this will also result in the shift of the peak position at 1310 nm. Therefore, this work proposes a simple and effective method of adjusting the thickness of the refractive index matching layer to achieve the above objectives (Figure. S4d).


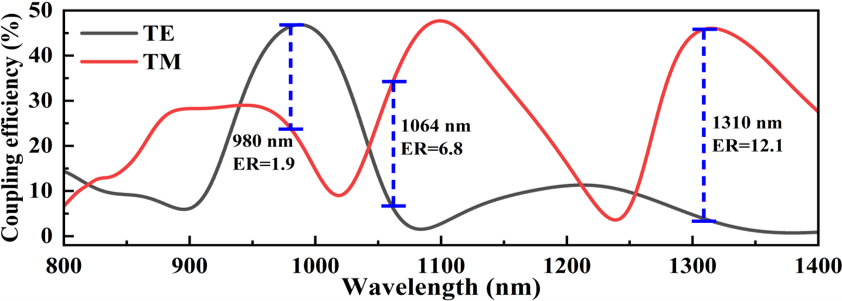


**Figure. S2.** The coupling efficiency of the grating coupler optimized solely by PSO algorithm at different wavelengths between TM and TE-polarization (*P* = 907.6 nm, *W* = 420 nm, *h* = 115.6 nm, *θ* = 75° and *T* = 868.5 nm).

As a comparison, for the device without refractive index matching layer (Figure. S3a), we also used the PSO algorithm to optimize coupling efficiency. The final result is shown in Fig. S3B, when the four optimized parameter values are *P* =1281 nm, *θ* =78.5°, *h* =159.9 nm, and *W* = 618 nm, approximately 44% of the coupling efficiency can be achieved at 1310 nm. The peak of coupling efficiency only appears at 1310 nm and TM-polarization.


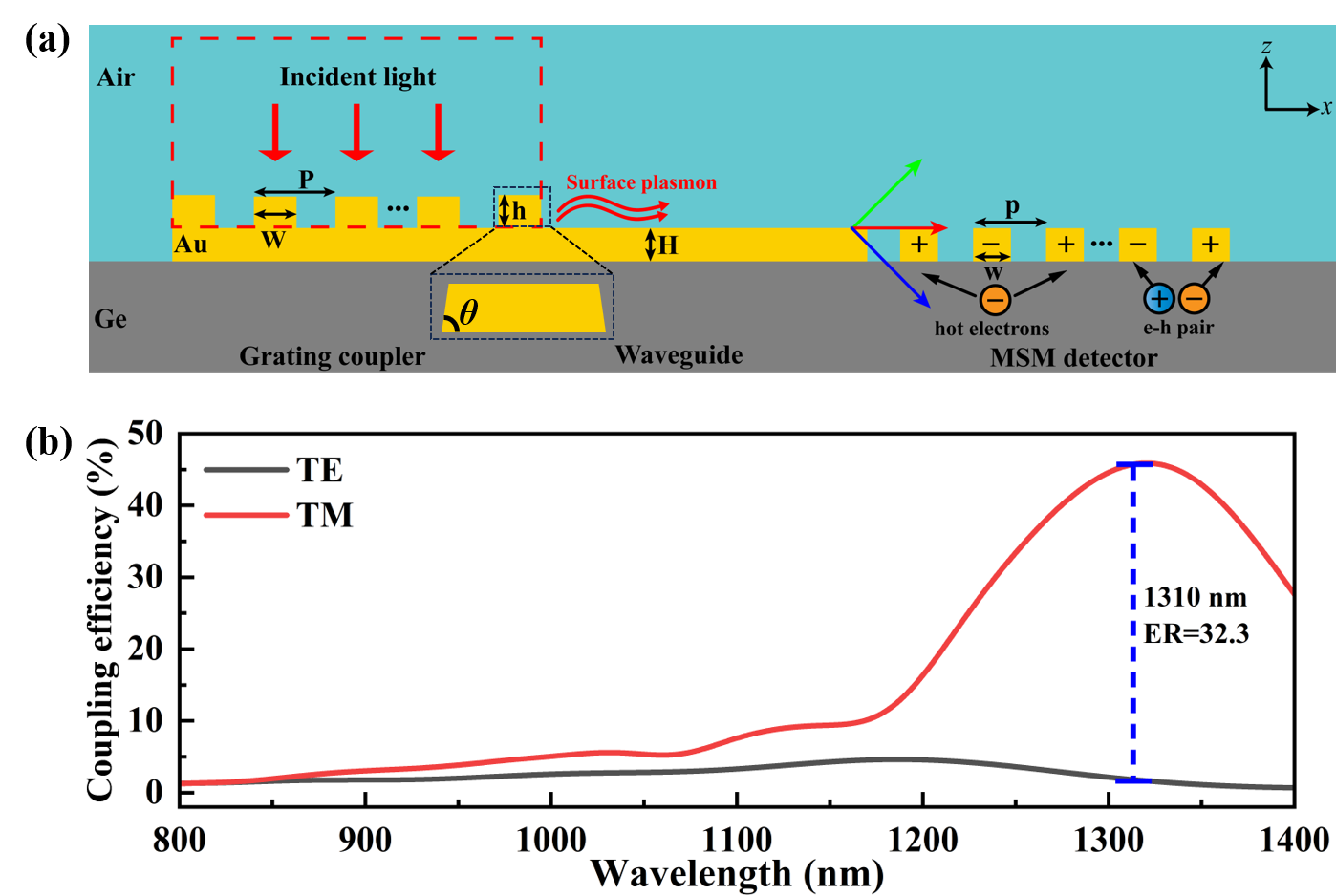


**Figure. S3.** (**a**) 2D structural diagram and working processes of the proposed plasmonic interconnect circuit without refractive index matching layer in *x-z* plane. Under the illumination of incident light (red straight arrow), local light field can be excited in the region of periodic coupling gratings (red dotted border). Partial light field then propagate along the waveguide (red curved arrow) and eventually outcouple to the air (green arrow), induce localized surface plasmon (red arrow), and interact with the Ge substrate (blue arrow) beneath the IDEs detector. (**b**) Coupling efficiency of the device without refractive index matching layer at different wavelengths between TM and TE-polarization (*P* =1281 nm, *θ* =78.5°, *h* =159.9 nm, and *W* = 618 nm).

**S2.** **Error tolerance of the grating coupler; Tunable effect of the refractive index matching layer.**

As mentioned in the main text, the pure SPP mode at 1310 nm has its optical field localized at the interface between the metal and the dielectric. Therefore, compared to the other two modes where the optical field is widely distributed in the dielectric layer, it is relatively less affected by the geometric changes in the dielectric layer. Therefore, it is feasible to adjust the peak position of the coupling efficiency dominated by other waveguide-strong-correlated optical modes by changing the thickness of the refractive index matching layer, without affecting the coupling efficiency of the designed pure SPP mode at 1310 nm. We believe that utilizing the correlations based on the different origins of optical modes to selectively adjust the operational characteristics of the device has significant implications for the design of other photonic devices as well. In Figure. S4d, when *T* is reduced from 868.5 nm to 800 nm, we can observe that there is only a slight change in the coupling efficiency peak at 1310 nm, while the peak and the line shape of the coupling efficiency at other wavelengths and polarization states both exhibit significant change. In addition, Figure S5 shows the variation of device decoupling efficiency with SiO_2_ *T*, which is highly consistent with the variation of coupling efficiency with *T*, this further proves that adjusting the peak position of optical modes by changing the thickness of the SiO_2_ layer is effective. At this time, as described in the main text, the higher coupling efficiency polarization extinction ratio can be achieved at 980 nm, 1064 nm, and 1310 nm (Figure. 2c). Additionally, we have scanned the effects of variations in other parameters on the coupling efficiency and found that the device exhibits good robustness within the range of process errors as shown in Figure. S4a-c. The tilt angle (*θ*) of the gratings is uncontrollable, but a tilt angle of 75° to 80° conforms to the actual process conditions, which coincides with what the optimization results.


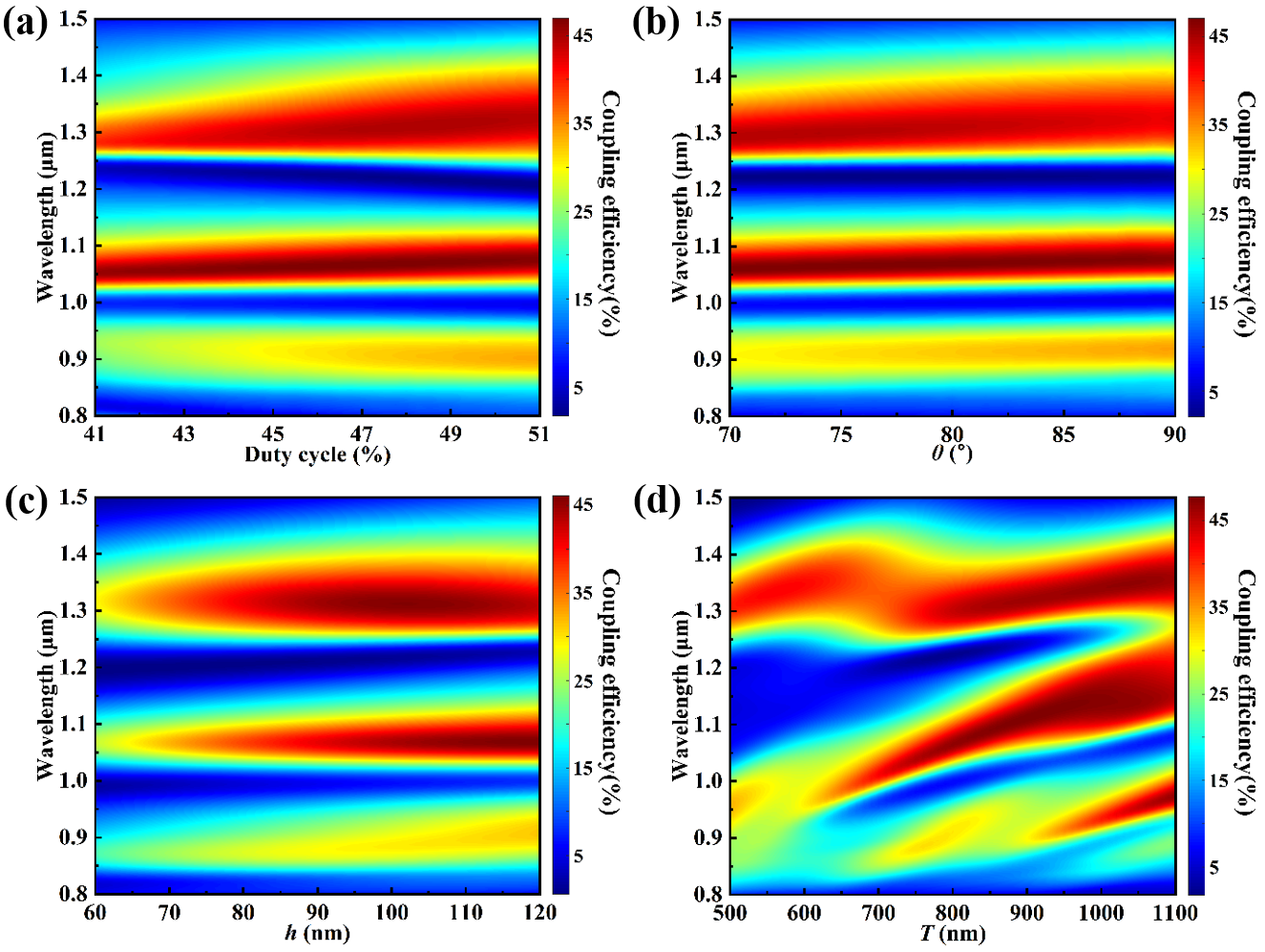


**Figure. S4.** The impact of changes in different coupling grating parameters on coupling efficiency at TM- polarization, (**a**) duty cycle, (**b**) tilt angle (*θ*), (**c**) the height of the top layer gratings (*h*), (**d**) the thickness of SiO_2_ refractive index matching layer (*T*).


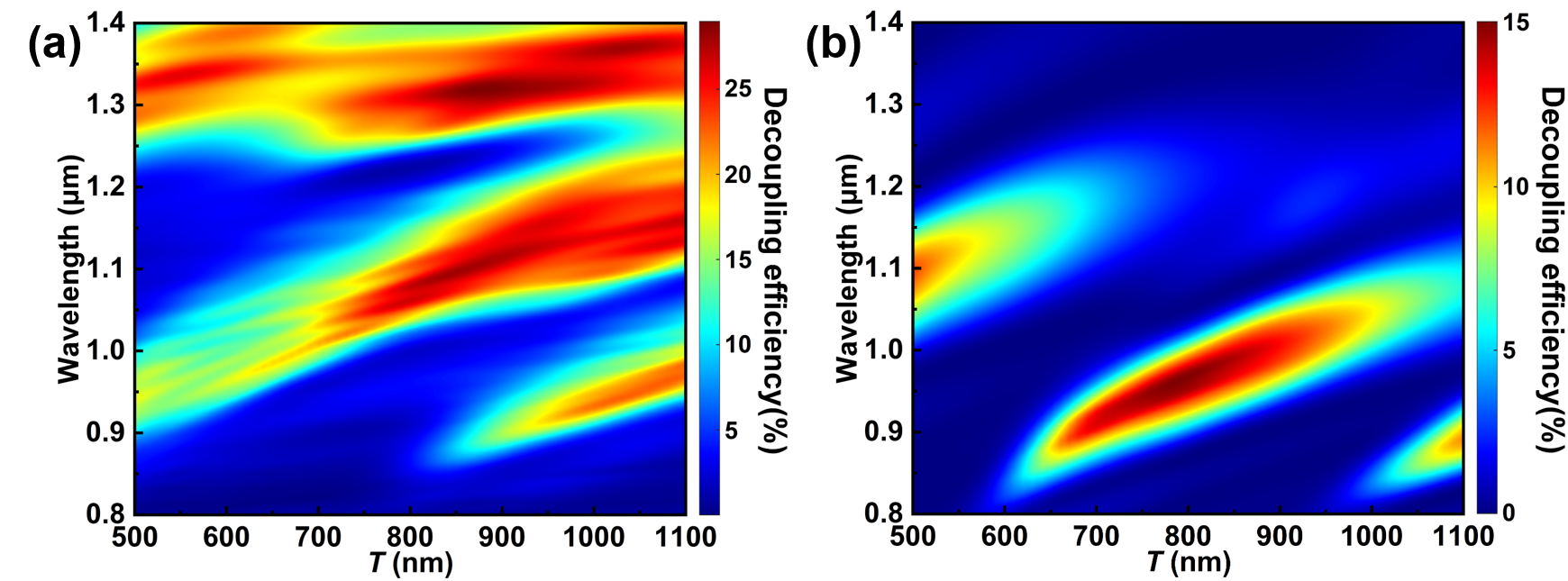


**Figure. S5.** The relationship between the decoupling efficiency and the thickness (*T*) of SiO_2_ film at (**a**) TM and (**b**) TE-polarization.

**S3.** **Optical band structure of coupling grating without refractive index matching layer; Coupling efficiency under different incident angles.**


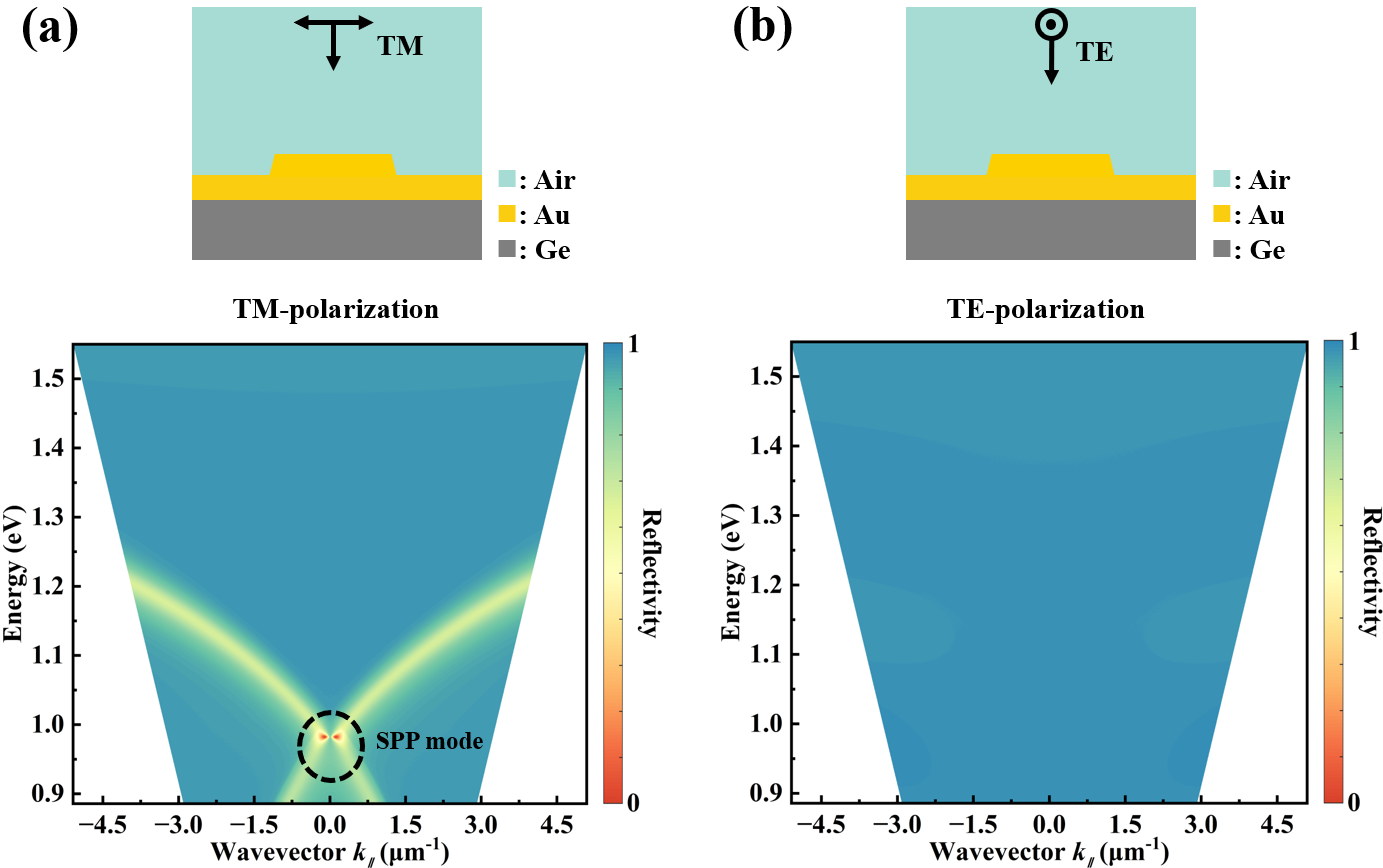


**Figure. S6.** Simulated optical band structure of the coupling grating without refractive index matching layer under (**a**) TM and (**b**) TE incident light.


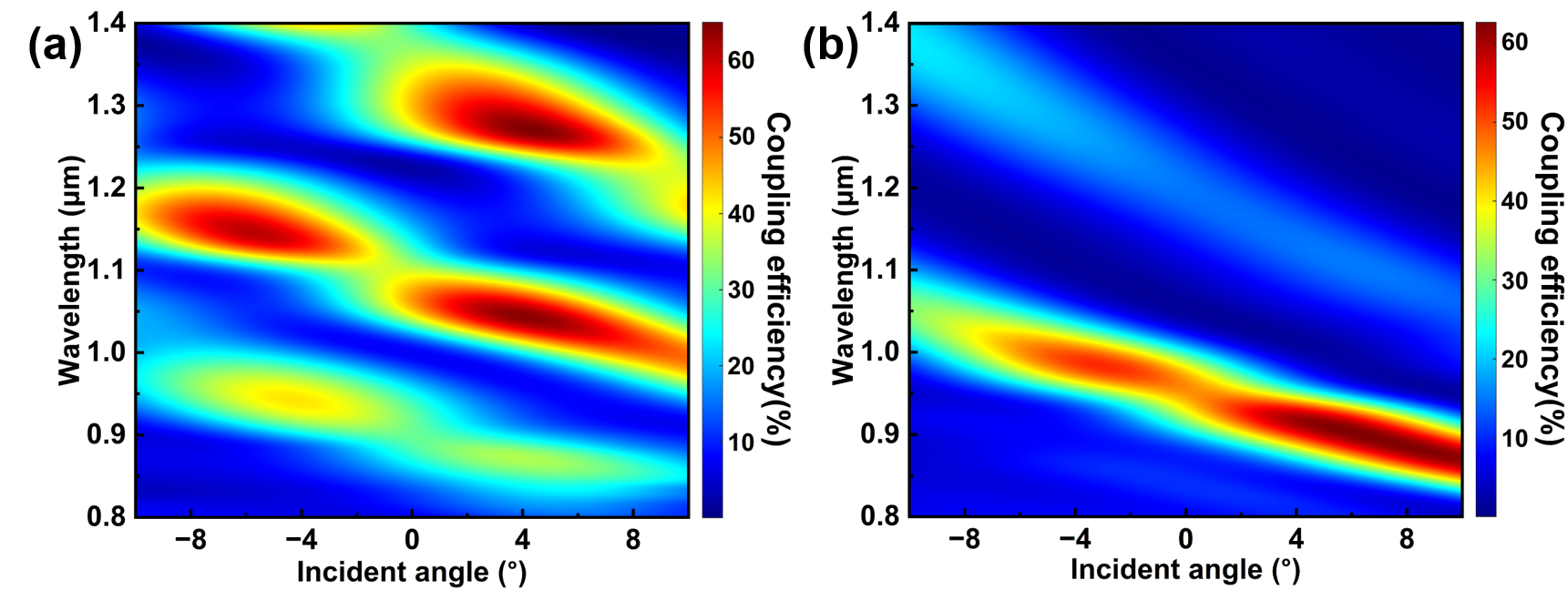


**Figure. S7.** Coupling efficiency of the device under different incident angles of illumination at (a) TM-polarization and (b) TE-polarization.

**S4.** **Device fabrication.**


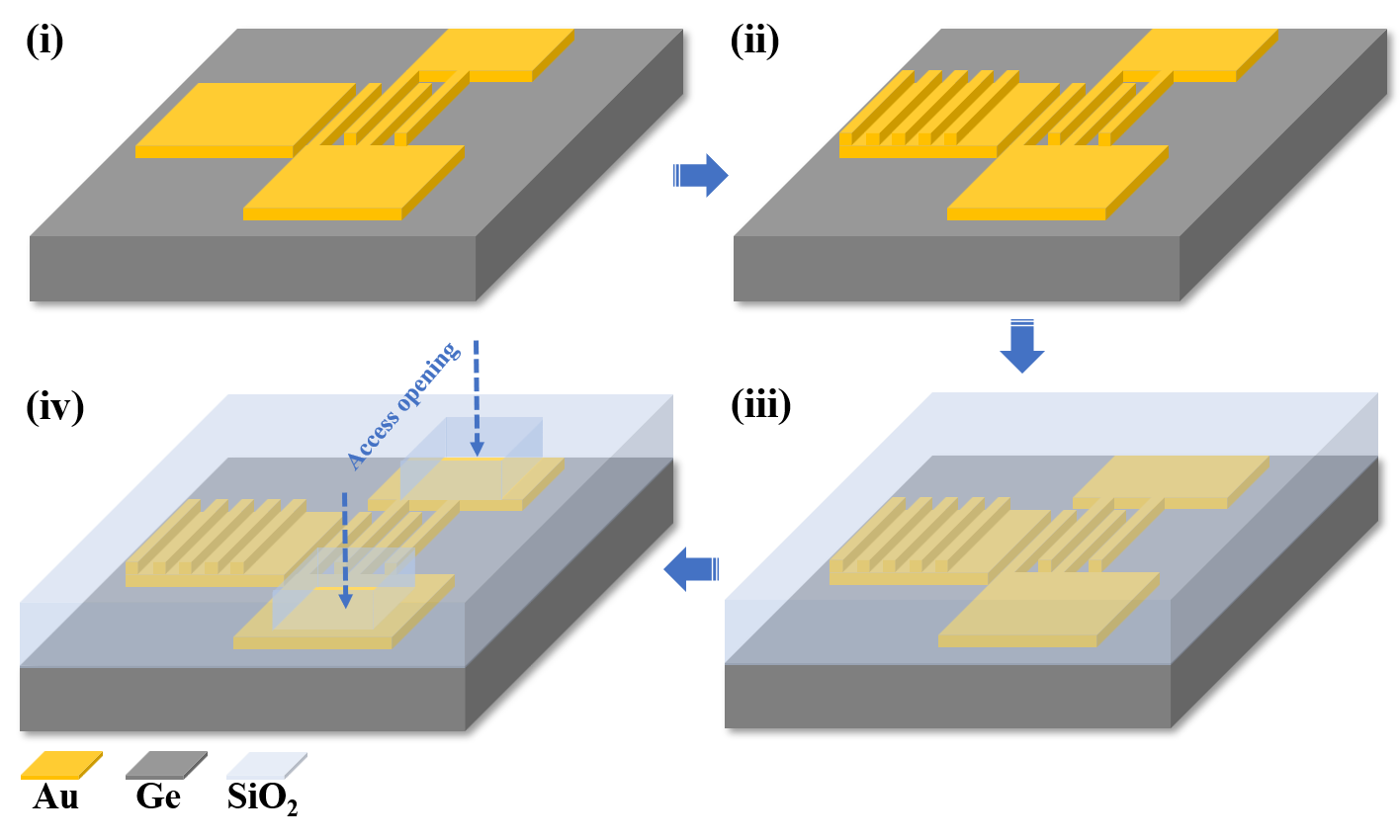


**Figure. S8.** Schematic of the core fabrication process for the device. (**i**) Fabricate the bottom metal layer, waveguide and IDEs. (**ii**) Fabricate the top coupling grating. (**iii**) Fabricate the SiO_2_ refractive index matching layer. (**iv**) Access opening on the contact pads.


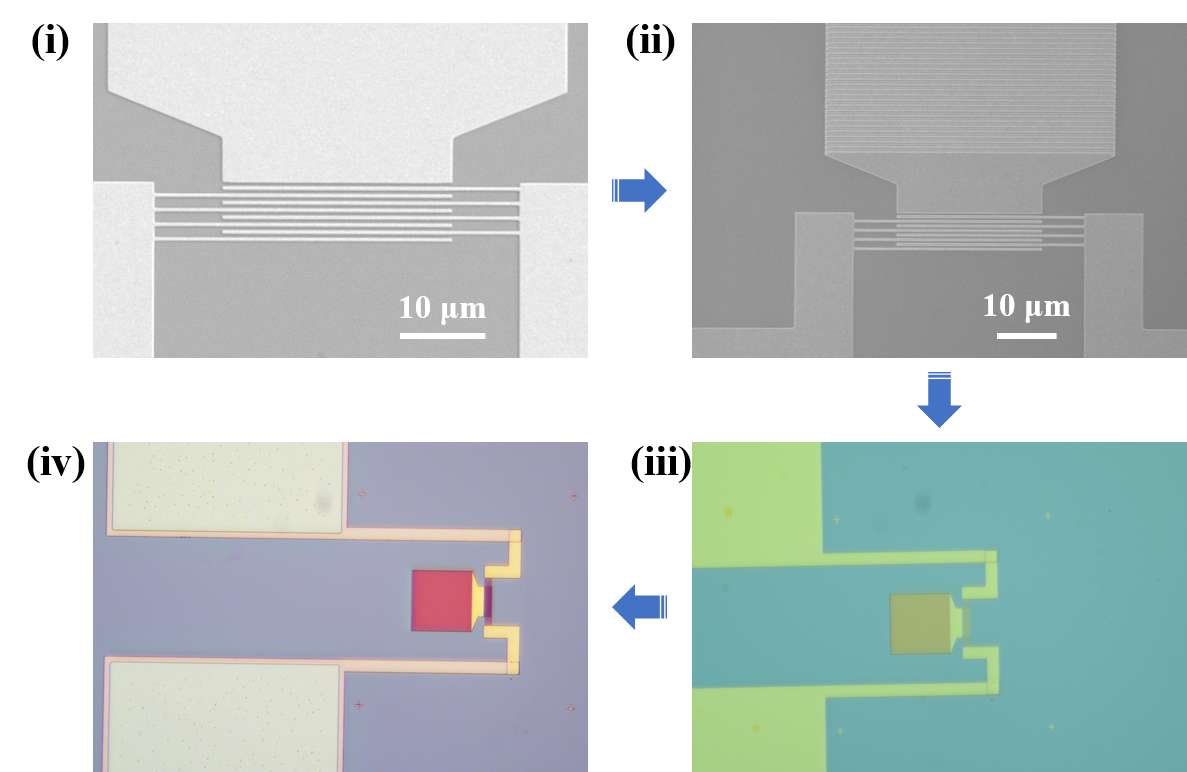


**Figure. S9.** SEM images and optical microscope images of the device at different stages of the fabrication process in Figure. S6. (Because SiO_2_ layer makes it difficult to take SEM images, optical microscopy images are used instead.)

**S5.** **Optoelectronic testing system.**


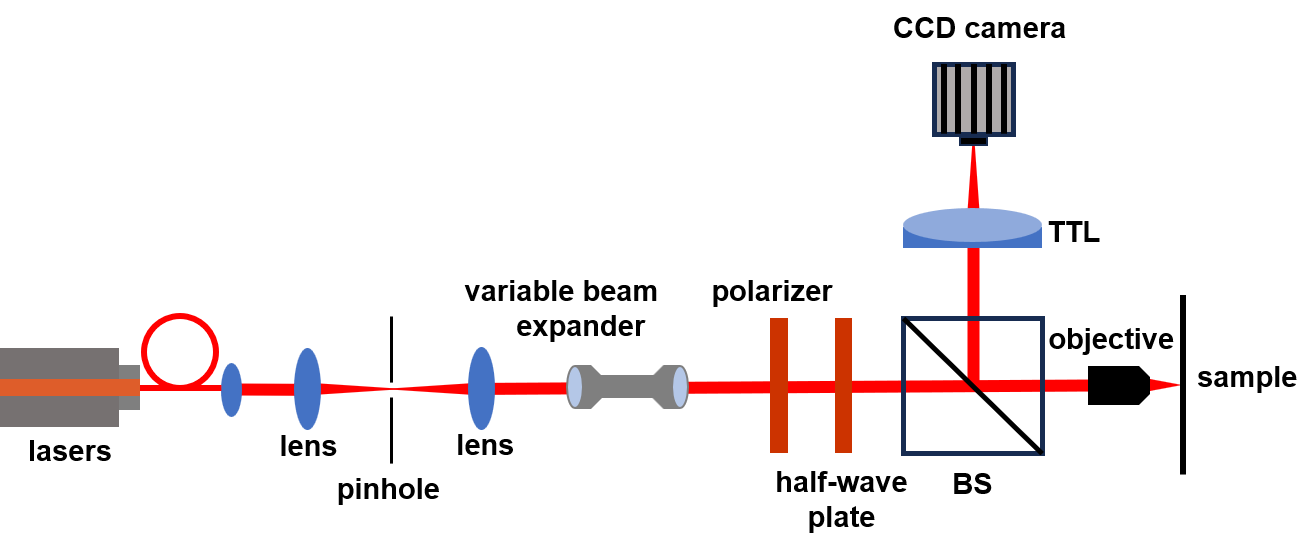


**Figure. S10.** Schematic diagram of the self-built optoelectronic measurement system.

**S****6. Basic performance of the MSM photodetector.**

The external quantum efficiency (EQE) can be expressed as:

$$\eta_{EQE}=\frac{I_{Electrons}\times1.24}{I_{Photons}\times\lambda(\mu m)}$$

*I_Electrons_* is the photocurrent passing through the detector under the illumination, 𝐼*_Photons_* is the intensity of the light on the grating coupler, and *λ* is the wavelength of the incident light. The photocurrent of the detector is equal to the value of the measurement current (with light) minus the dark current.


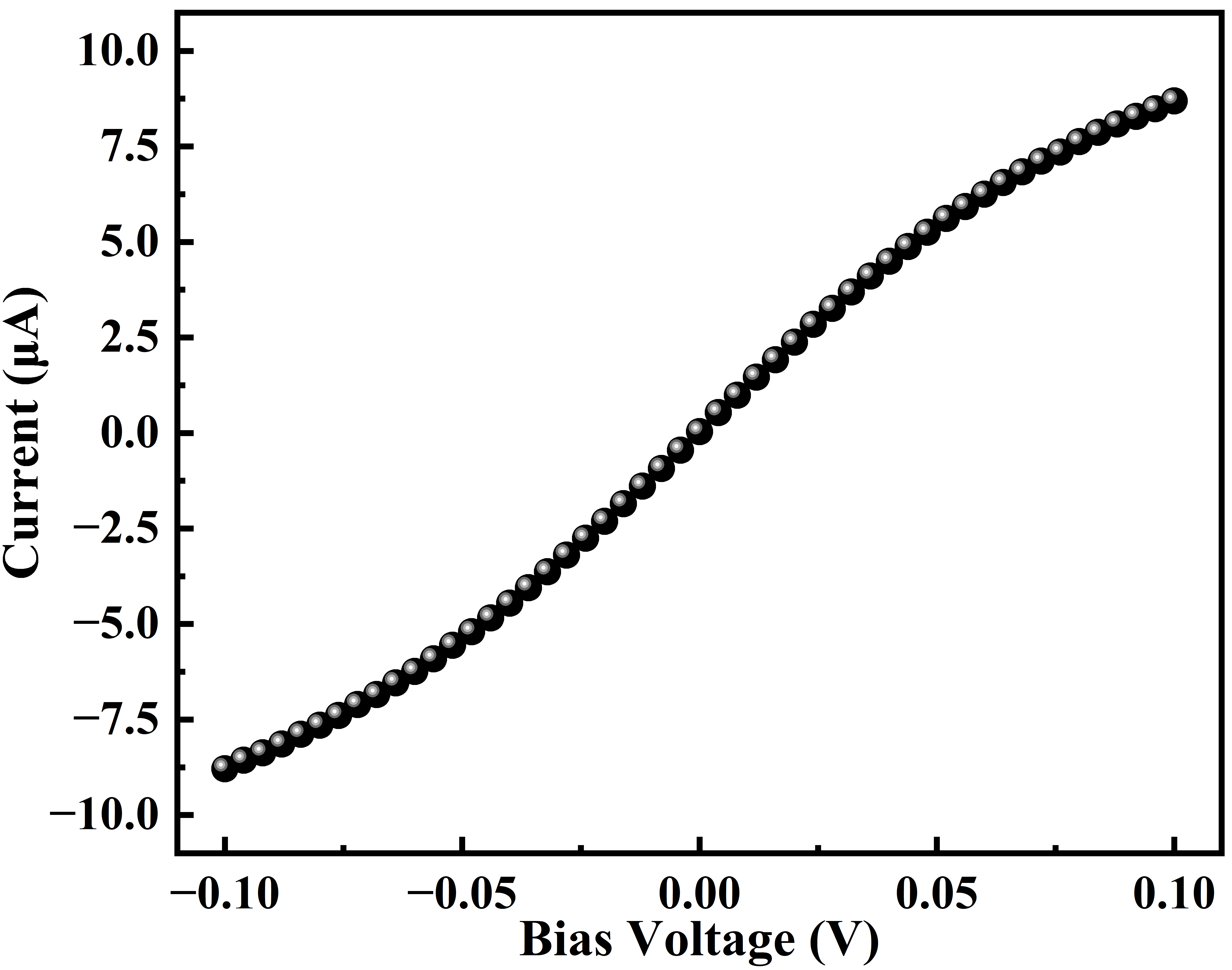


**Figure. S11.** The dark *I*–*V* characteristic of the prepared plasmonic interconnect circuit.


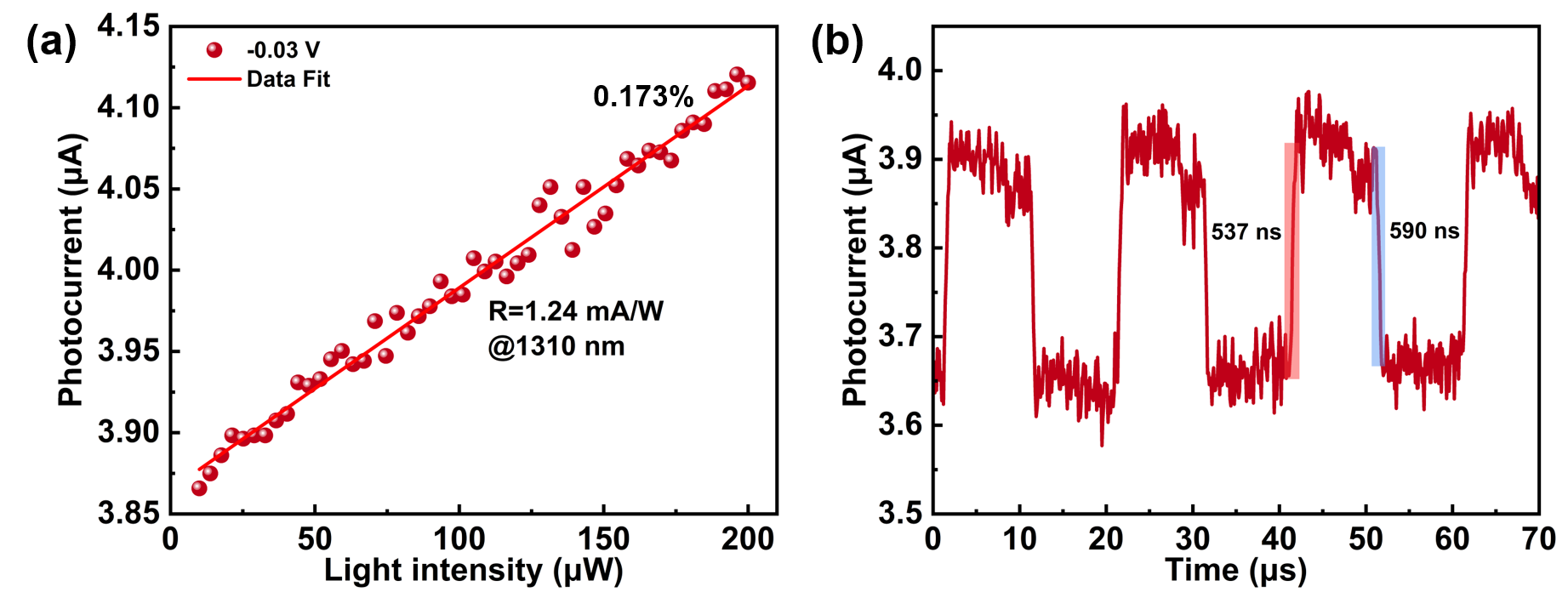


**Figure. S12.** (**a**) Photocurrent as a function of the input light power at 1310 nm, (**b**) Response speed of the photodetector at 1310 nm.

**S7. Polarization extinction ratio of the decoupling efficiency at different SiO_2_ thicknesses.**


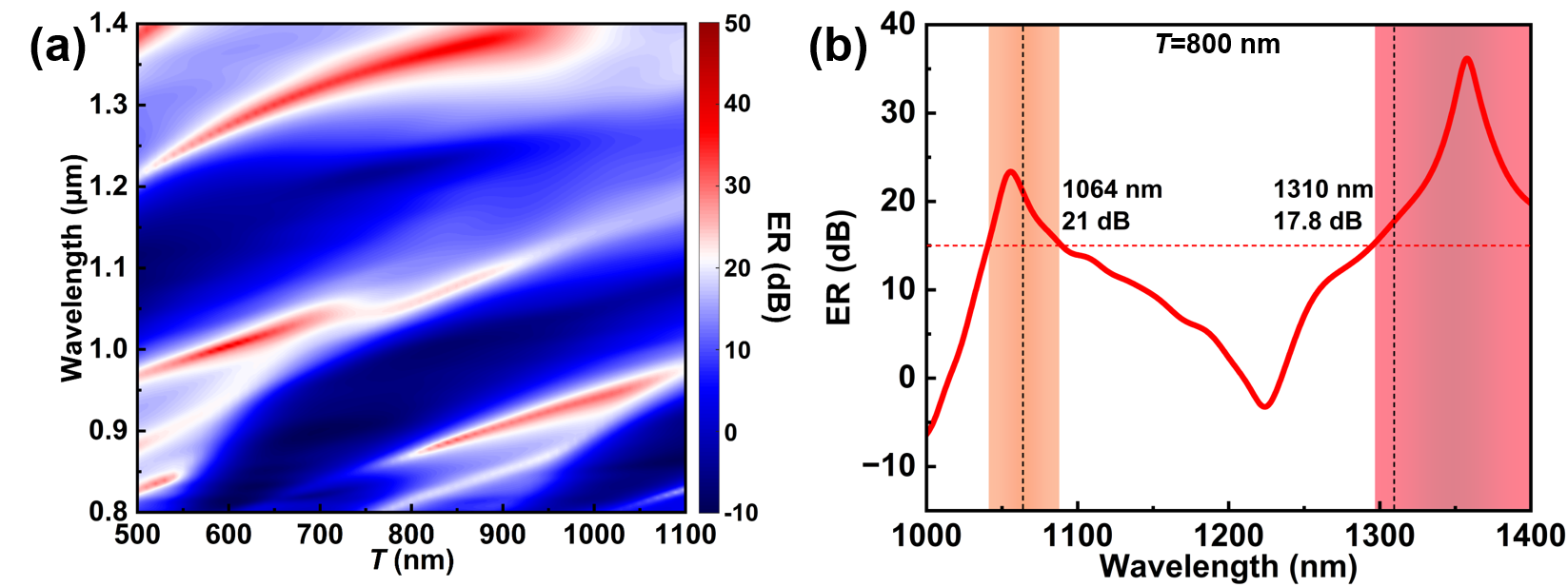


**Figure. S13.** (**a**) Polarization extinction ratio of the decoupling efficiency at different SiO_2_ thicknesses, expressed in decibels (TM/TE), (**b**) Polarization extinction ratio of SiO_2_ layer with 800 nm thickness, where the colored region has an extinction ratio greater than 15 dB.
